# Systems‐Level Plant Responses Reveal *Pseudomonas*‐Mediated Growth Promotion in *Brachypodium* Under Nitrogen Limitation

**DOI:** 10.1111/pce.70615

**Published:** 2026-05-25

**Authors:** Stefan Sanow, Melissa Mantz, Holger Wissel, Atul Bhatnagar, Elena Sturm, Jana M. Kelm, Robert Walker, Andreas Lücke, Gabriel Schaaf, Pitter F. Huesgen, Ute Roessner, Michelle Watt, Borjana Arsova

**Affiliations:** ^1^ Institute for Bio‐ & Geosciences, Plant Sciences (IBG‐2) Forschungszentrum Jülich GmbH Jülich Germany; ^2^ School of BioSciences, Faculty of Science The University of Melbourne Parkville Victoria Australia; ^3^ Institute of Engineering, Electronics and Analytics, Analytics (ZEA‐3) Forschungszentrum Jülich GmbH Jülich Germany; ^4^ Faculty of Biology University of Freiburg Freiburg Germany; ^5^ Institute for Bio‐ & Geosciences, Agrosphere (IBG‐3) Forschungszentrum Jülich GmbH Jülich Germany; ^6^ Sydney Mass Spectrometry, DVC Research The University of Sydney Sydney New South Wales Australia; ^7^ VCCC The University of Melbourne Parkville Victoria Australia; ^8^ Institute of Crop Science and Resource Conservation University of Bonn Bonn Germany; ^9^ CIBSS – Centre for Integrative Biological Signalling Studies University of Freiburg Freiburg Germany

**Keywords:** biological nitrogen fixation, cereals, lipidomics, phenotyping, plant growth–promoting bacteria, plant–microbe interaction, proteomics

## Abstract

Plant growth–promoting bacteria can enhance plant performance under nutrient limitation, yet the underlying plant molecular responses remain incompletely resolved. We investigated growth promotion by *Pseudomonas koreensis* in *Brachypodium distachyon* under contrasting nitrogen (N) regimes using time‐resolved phenotyping, elemental analysis, lipidomics and proteomics. Shoot phenotyping revealed rapid responses to N availability, whereas beneficial effects of bacterial inoculation emerged only during prolonged growth under low N. Under N limitation, inoculated plants accumulated significantly more biomass and total N than uninoculated controls, reaching levels comparable to high N plants, while no inoculation effect was observed under high N. Biomass increases were accompanied by only modest changes in tissue N concentration, indicating enhanced whole‐plant N‐use efficiency rather than disproportionate N enrichment. Proteomics identified N availability as the primary determinant of proteome structure, with bacterial inoculation under low N conditionally modulating selected modules towards High N states. Lipidomic profiles were largely N‐driven, with only transient inoculation effects at early stages. Despite the presence of N fixation–associated genes in *P. koreensis*, δ^15^N analyses did not support substantial in planta N fixation. Together, these results support a plant‐centric model in which bacterial inoculation enhances growth under N limitation by modulating plant‐encoded N acquisition and metabolic organization within an N‐defined framework.

## Summary statement

The plant growth–promoting bacterium *Pseudomonas koreensis* improves *Brachypodium distachyon* growth under limited nitrogen by modulating plant‐encoded N acquisition and metabolic organization within an N‐dependent metabolic framework.

## 1 Introduction

Agriculture increasingly requires biological strategies that can reduce mineral fertilizer inputs while maintaining crop productivity and minimizing environmental impacts. One promising approach involves the use of plant growth–promoting bacteria (PGPB), which can enhance plant performance by improving nutrient availability, modulating plant physiology or both. However, the efficacy of PGPB is often highly context‐dependent, varying with environmental conditions such as nutrient availability, soil properties and plant developmental stage, which limits their consistent application in agricultural systems (Lopes et al. 2021).

Dissecting how plants respond to beneficial microbes under defined abiotic conditions is therefore critical to understand the mechanisms underlying PGPB‐mediated growth promotion. Recent work using *Brachypodium distachyon* demonstrated that inoculation with *Herbaspirillum seropedicae* resulted in distinct growth responses under contrasting nitrogen (N) regimes, involving differences in nitrogen uptake rates, nitrogen forms utilized, transcriptional regulation and temporal dynamics (Kuang et al. 2022). These findings highlight that plant responses to beneficial microbes are not uniform but are shaped by nutrient context. Nevertheless, how such interactions are organized at the system level, integrating metabolism, regulation and resource allocation, remains poorly understood.

Plant–microbe interactions trigger complex and coordinated responses involving signalling proteins, lipids and metabolites. While transcriptomic and proteomic studies have provided insights into regulatory and metabolic adjustments, the contribution of lipid metabolism to plant responses during beneficial microbial interactions has received comparatively little attention. Recent work has begun to highlight lipid remodelling as an important component of plant–microbe interactions and stress adaptation (Schillaci et al. 2021), underscoring the need for integrated, multi‐omics approaches to capture the plasticity of plant molecular responses.

The genus *Pseudomonas* is globally distributed and includes species that range from pathogenic to beneficial, with many strains capable of enhancing plant performance under sub‐optimal nutrient conditions (Preston 2004; Volke et al. 2020; Rooney et al. 2020; M. Wang et al. 2020; Sanow et al. 2023; Singh et al. 2024). Beneficial *Pseudomonas* spp. have been reported to influence plant nitrogen nutrition through multiple, potentially interacting mechanisms, including modulation of nitrogen availability, production of secondary metabolites and interactions with other members of the microbial community (Sah et al. 2021; Tu et al. 2021). However, the relative importance of these processes in planta, and their dependence on nitrogen availability, remains unresolved.

*P. koreensis* Ps 9‐14^T^ was originally isolated from Korean soils (Kwon et al. 2003) and has since been associated with plant‐beneficial traits, including indirect disease suppression through effects on microbial community composition (Han et al. 2021). A related strain, *P. koreensis* IB‐4, has been proposed as a biocontrol agent in potato, and genomic analyses indicate the presence of genes associated with nitrogen fixation, with nitrogenase activity detected under laboratory conditions (Rafikova et al. 2016). Whether such genetic potential translates into increased plant nitrogen acquisition in planta, however, has not been demonstrated.

Here, we investigated how *B. distachyon* responds to inoculation with *P. koreensis* Ps 9‐14^T^ under contrasting nitrogen regimes. By integrating time‐resolved phenotyping, elemental analysis, proteomics and lipidomics, we aimed to characterize plant physiological and molecular responses to bacterial inoculation within a nitrogen‐defined context. Proteomic profiling provided insights into whole‐plant protein abundance patterns, while lipidomics enabled the examination of dynamic changes in lipid metabolism, a largely unexplored dimension of beneficial plant–microbe interactions (Macabuhay et al. 2022). Rather than testing a single mechanistic hypothesis, this study uses *P. koreensis* as a model PGPB to examine how nitrogen availability structures plant responses to microbial presence and to evaluate the potential of this interaction to improve growth in grass species, including crops of global importance.

## 2 Material and Methods

### 2.1 Plant Cultivation Conditions

*B. distachyon* Bd21‐3 (Vogel and Hill 2008) seeds were surface sterilized (Sasse et al. 2019), dehusked and sown into modified Plant Cultivation Vessels (PCVs; GA‐3, Kisker Biotech; Supporting Information S9: Figure 2) filled with 900 g of 0.7–1.4 mm quartz sand. Containers (except connectors) were autoclaved and oven‐dried. In total, 54 g of modified Hoagland solution (1.76 mL 0.5 M KH_2_PO_4_, 2.64 mL 1 M MES (pH 5.7), 4.39 mL 10 mM FeSO_4_ + 10 mM Na_2_EDTA, 1.76 mL 1 M CaCl_2_, 1.76 mL 0.375 M MgSO_4_, 1.76/17.6 mL 1 M NH_4_NO_3_ (low/high N) and 0.44 mL micronutrients (50 mM H_3_BO_3_, 10 mM MnSO_4_, 2 mM ZnSO_4_, 5 mM KI, 0.2 mM Na_2_MoO_4_, 0.1 mM CoCl_2_, 1 mM CuSO_4_) in a total volume of 1 L) was added to each under sterile conditions.

Three sowing holes were prepared using a template (Supporting Information S9: Figure 3). One seed was placed per hole under sterile conditions and covered with sand. At 5 days after sowing (DAS), excess seedlings were removed to ensure uniform shoot height.

Three independent experiments were conducted, each comprising four treatments: Low N, Low N + Pk, High N and High N + Pk (Supporting Information S9: Figure 4).

### 2.2 Bacterial Cultivation and Inoculation

*P. koreensis* Ps 9‐14^T^ (Pk) (DSM16610, Germany) was cultivated using Luria‐Bertani (LB) medium at 29°C and 220 rpm for liquid culture. For solid cultures, LB medium was supplemented with 1% (w/v) agar. Overnight liquid cultures were used for plant inoculation as described below. Liquid cultures were used exclusively for inoculation experiments, whereas agar plates were used only for routine cultivation and colony maintenance.

A pre‐recorded bacterial growth curve was established with an OD_600_ of 0.5 corresponding to 1 × 10^8^ CFU mL^−1^. Bacteria from the overnight culture were centrifuged at 3000 rpm for 15 min and the bacterial pellet was resuspended in 1 mL of the respective Hoagland solution and further diluted to 5 × 10^6^ CFU mL^−1^. Each seedling received 100 µL inoculum (5 × 10^5^ CFU); controls received media. PCVs were imaged for non‐invasive shoot phenotyping (cf. non‐invasive shoot phenotyping) before being closed and transferred to growth chambers.

### 2.3 Plant Growth Conditions and Experimental Setup

**Figure 1:**
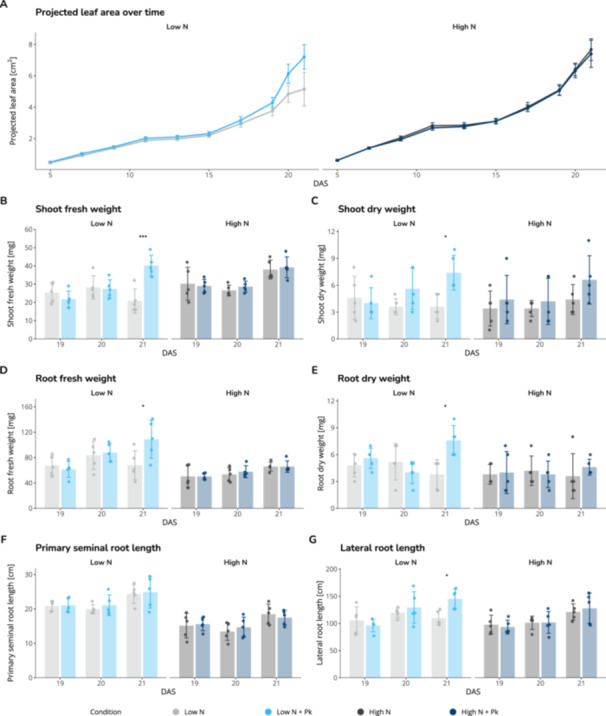
Nitrogen‐dependent growth responses to Pk inoculation. (A) Time series of projected leaf area (PLA) measured non‐invasively from 5 to 21 (DAS). Values represent means ± 95% confidence intervals. Plants were imaged repeatedly over time; sample size declined at later time points due to destructive harvests (up to and including 19 DAS *n* = 33 plants per treatment; *n* = 22 at 20 DAS and *n* = 11 at 21 DAS). (B and C) Shoot fresh weight (FW) and shoot dry weight (DW). (D and E) Root fresh weight (FW) and root dry weight (DW). (F) Primary seminal root length (PSRL). (G) Lateral root length (LRL). Destructive traits (B–G) were measured at 19, 20 and 21 DAS (*n* = 5 biological replicates per treatment and time point). Points represent individual plants; bars indicate means ± SD. Statistical comparisons for destructive traits (B–G) were performed separately for each time point using two‐way ANOVA (nitrogen × inoculation) followed by Tukey's HSD; asterisks indicate Tukey‐adjusted differences between Pk and Ctrl within each nitrogen condition (*p* < 0.05; *p* < 0.01; *p* < 0.001).

Plants were cultivated for 21 DAS in growth chambers (Percival Scientific, Model AR‐22L) under a 16 h light/8 h dark photoperiod at 23°C/18°C, 100–140 µmol m^−2^ s^−1^ light intensity, and 35%–40% relative humidity. PCVs were distributed across three identical chambers and randomized at each imaging time point. Plants were removed from chambers at 5 DAS for inoculation and subsequently imaged every 2 days; watering occurred at 7 and 14 DAS (Supporting Information S9: Figure 1).

Across three independent experiments, non‐invasive phenotyping included 44 plants per treatment. Destructive phenotyping and elemental analyses were performed using five biological replicates per treatment and time point. Lipidomics used six biological replicates per treatment and time point. Proteomics was conducted with four to five biological replicates per tissue and treatment, each consisting of pooled material from two plants.

### 2.4 Non‐Invasive Shoot Phenotyping

Shoots were imaged from three angles using a smartphone (Samsung Note8) in a beige‐background photobox inside a sterile hood (Supporting Information S9: Figure 5). Projected leaf area (PLA) was calculated via HSV colour segmentation (Müller‐Linow 2022) with thresholds: Hue 44.4–127.5, Saturation 66.6–255, Value 6.75–255. The shoot phenotyping interval was: 5, 7, 9, 11, 13, 15, 17, 19, 20, 21 DAS. Invasive harvests were conducted at 19, 20, 21 DAS (Supporting Information S9: Figure 4).

### 2.5 Invasive Root and Shoot Phenotyping

A total of 44 plants were harvested at each 19, 20, 21 DAS. Five plants per time point and treatment were used for invasive phenotyping and six plants for molecular analyses.

Roots were washed with sterile Milli‐Q water and separated from shoots. Fresh weight (FW) was recorded for both tissues immediately after harvest. For morphological analysis, root systems were scanned freshly using a flatbed scanner and the WinRhizo software (Regent Instruments) prior to any further processing. For molecular analyses, root and shoot tissues were flash‐frozen in liquid N_2_ immediately after FW determination and stored at −80°C until protein or lipid extraction. Samples were oven‐dried at 65°C for 3 days, weighed and homogenized (Retsch MM400, 3 mm bead, 30 Hz, 1 min) for C/N analysis (Cario EL Cube, Elementar) with thermal conductivity detection. Root images generated with WinRhizo were analyzed using PaintRhizo (Nagel et al. 2012) to quantify total root length and to distinguish primary, seminal and lateral root length.

### 2.6 Sample Preparation for Protein Extraction

Roots and shoots from two plants were pooled per biological replicate (≥ 100 mg roots; ~55 mg shoots), four to five biological replicates were used per tissue (root or shoot) and treatment. From each, 30–50 mg was homogenized (ball mill, 5 × 1 min) without thawing. Proteins were extracted with PBS + 2% SDS (100 µL per 10 mg FW) and quantified via BCA assay. For digestion, 25 µg protein was denatured (10 mM DTT, 90°C, 15 min), alkylated (50 mM chloroacetamide, 30 min dark, room temperature) and quenched (50 mM DTT, 20 min). SDS removal and trypsin digestion (1:100) followed the SP3 protocol (Demir et al. 2022), with overnight incubation at 37°C. Peptides were cleaned using modified SDB‐RP StageTips (Rappsilber et al. 2007), eluted, vacuum‐concentrated and resuspended in 0.1% formic acid. In total, 2 μL of the sample were used to determine peptide concentration at the Nanodrop, before injection of 1 µg peptides into LC–MS/MS.

### 2.7 Proteomics Data Acquisition

In total, 1 µg peptides per sample was analyzed via LC–MS/MS using an UltiMate 3000 RSLC nano‐HPLC system (Thermo) with μPAC pillar array trap and analytical columns (1 cm and 50 cm flowpath, respectively, PharmaFluidics) on‐line coupled to an Impact II Q‐TOF mass spectrometer (Bruker) with CaptiveSpray source (Bruker) as described (Beck et al. 2015). Peptides were separated using an 80 min 5%–32.5% ACN gradient (600 nL/min, 40°C) and tandem mass spectrometry data were acquired using a data independent acquisition (DIA) scheme with MS1 survey scans from 400 to 1200 m/z followed by acquisition of MS2 fragment ion in 32 selection windows of 25 m/z with 0.5 m/z overlap, scanning a mass range from 200 to 1750 m/z at 15 Hz.

### 2.8 Proteomics Data Analysis and Visualization

Peptides were identified by matching DIA data to a predicted spectral library using DIA‐NN v1.8 (Demichev et al. 2020) against *B. distachyon* protein sequences from UniProt (proteome UP000008810_15368, release 2022_04). Trypsin was specified as the digestion enzyme with up to two missed cleavages allowed. Carbamidomethylation of cysteine was set as a fixed modification and oxidation of methionine as a variable modification. Precursor false discovery rate (FDR) was controlled at 1%. Peptides with lengths of 7–30 amino acids and precursor charge states of 2–4 were considered. The analysis was performed in double‐pass mode with match‐between‐runs enabled and heuristic protein interference disabled.

Functional annotation of protein sequences was performed using Mercator4 v2.0 (Schwacke et al. 2019). Quantitative proteomics data were further processed in Perseus v1.6.15.0. Differential protein abundance analyses are described in Section 2.11. Proteins and modules of interest were visualized using MapMan v3.5.1R2 on modified nitrogen metabolism maps (Arsova et al. 2012; Feng et al. 2020). Kinase family and lipid biosynthesis maps were obtained directly from the MapMan website.

Weighted gene co‐expression network analysis (WGCNA) was performed in R as described below.

### 2.9 Confirmation of Plant Inoculation

The presence of *P. koreensis* in inoculated samples was confirmed by MaxQuant searches against a *P. koreensis* reference proteome (UP001139955, release 2023_04) using identical search parameters.

### 2.10 LC–MS Untargeted Analysis for Lipids

Root tissue (~20 mg FW) was extracted following Kehelpannala et al. (2021) and dried in vacuo (Christ Alpha 2‐4 LSCplus). Lipid extracts were reconstituted in 100 µL butanol:methanol (1:1, v/v) containing 1% ammonium acetate, vortexed, shaken (20°C, 1400 rpm, 10 min) and centrifuged (5 min, 13 000 rpm, room temperature). Aliquots (7 µL) from each sample were combined to generate a pooled biological quality control (PBQC) sample, which was injected periodically throughout the analytical sequence to monitor instrument performance.

Lipidomic data were acquired using a high‐resolution Q‐TOF mass spectrometer (SCIEX TripleTOF 6600) coupled to an ExionLC UHPLC system, operating in sequential window acquisition of all theoretical mass spectra (SWATH) mode. The total runtime was 30 min per sample at a flow rate of 0.26 mL min^−1^. Gradient conditions followed Kehelpannala et al. (2021). Source parameters were set as follows: curtain gas 35, ion source gases 1 and 2 at 25, temperature 250°C and ion spray voltage 4500 V. Mass ranges were 100–1700 m/z for both MS1 and MS/MS scans, with SWATH windows of 16.2 Da. Data were processed using MS‐DIAL v5.1.230719 (Tsugawa et al. 2015). Six independent biological replicates were analyzed per treatment and time point. Lipidomics data have been deposited in MetaboLights under accession MTBLS12763.

Supporting Information S9: S9 section contains information on: ^15^N natural abundance and enrichment analysis; Growth of bacteria on nitrogen‐deficient media and quantitative real‐time PCR, all of which are Supporting Information to the data presented here.

### 2.11 Data Analysis and Statistics

#### 2.11.1 Phenotypic, Biomass and Elemental Analyses

Effects of nitrogen availability (low vs. high) and bacterial inoculation (*P. koreensis* vs. control) on phenotypic traits, root architecture, biomass and elemental composition were assessed using two‐way analysis of variance (ANOVA) models with nitrogen availability and inoculation as fixed factors. When significant main effects or interactions were detected, pairwise comparisons were performed using Tukey's honestly significant difference (HSD) tests. Significance was assessed at *p* < 0.05 unless stated otherwise. Sample sizes are indicated in the corresponding figure legends.

Data are presented as means ± standard deviation (SD) unless otherwise indicated. For time‐series and elemental measurements, uncertainty is shown as 95% confidence intervals (CI), whereas destructive traits with individual data points are presented as means ± SD.

#### 2.11.2 Lipidomics

##### 2.11.2.1 Data Processing

Lipid features were filtered based on retention time (1.5–25 min), signal‐to‐noise ratio (> 5) and reproducibility across PBQC samples (coefficient of variation < 25%). Feature abundances were log_10_‐transformed; non‐finite and non‐positive values were replaced with half of the minimum positive value prior to transformation. Lipid features were Pareto scaled (mean‐centred and divided by the square root of the SD).

##### 2.11.2.2 Multivariate Analysis

Global lipidomic patterns were explored using principal component analysis (PCA) and hierarchical clustering analysis (HCA) for visualization purposes. PCA was performed separately at 19, 20 and 21 DAS using the prcomp function in R on centred data without additional scaling. Heatmaps were generated from per‐feature *z*‐scores, averaged within each condition and clustered by lipid species using Pearson's correlation distance and average linkage.

##### 2.11.2.3 Differential Abundance Testing

Treatment effects on individual lipid features were assessed using two‐way ANOVA with nitrogen availability and *P. koreensis* inoculation as factors, followed by Tukey's HSD post hoc tests. No global multiple‐testing correction was applied for lipidomics; statistical results were interpreted conservatively and in conjunction with multivariate analyses, given the exploratory nature of the data set and the modest effect sizes observed.

#### 2.11.3 Proteomics

##### 2.11.3.1 Multivariate Analysis

PCA was performed on proteins quantified with 100% valid values across all treatments. Protein abundances were log_10_‐transformed and Pareto scaled per feature. PCA was computed using the prcomp function in R on centred data without additional scaling.

##### 2.11.3.2 Differential Abundance Analysis

Differential protein abundance analyses were performed on proteins quantified across all biological replicates in all conditions (100% valid values). Pairwise differences between treatment conditions were assessed using Tukey's HSD tests. To control FDRs across proteins for each pairwise comparison, Tukey *p* values were adjusted using the Benjamini–Hochberg procedure. FDR‐adjusted *q* values (tukey_q_fdr < 0.05) were used to define statistically significant pairwise differences. Permutation‐based *q* values from Perseus are reported for reference but were not used to define contrast‐specific significance.

#### 2.11.4 Co‐Expression Network Analysis (WGCNA)

A protein abundance matrix was constructed from quantitative proteomics data and filtered to retain proteins quantified in at least half of all samples. Missing values were imputed per protein using half of the minimum observed value. WGCNA was performed using an unsigned network with a soft‐thresholding power of 6. Modules were defined with a minimum size of 10 proteins and merged at a cut height of 0.25 (Langfelder and Horvath 2008).

Module–trait associations were calculated as Pearson's correlations between module eigengenes and experimental variables (nitrogen availability, *P. koreensis* inoculation and their interaction). Corresponding *p* values were adjusted for multiple testing across modules using the Benjamini–Hochberg procedure.

To assess *P. koreensis* effects within nitrogen backgrounds, module eigengenes were additionally modelled using linear models with nitrogen, inoculation and their interaction as predictors. Simple effects of inoculation within each nitrogen level were estimated using the emmeans package, and resulting *p* values were adjusted across modules within each nitrogen stratum using Benjamini–Hochberg correction.

## 3 Results

### 3.1 *Pseudomonas koreensis* Inoculation Enhances Growth and Biomass Accumulation Under Nitrogen Limitation

Non‐invasive time‐series analysis of PLA showed that plant growth was primarily determined by nitrogen availability until 15 DAS (Figure 1A). Under low nitrogen, Pk‐inoculated plants began to diverge from uninoculated controls at 17 DAS, with a pronounced increase in PLA at later stages. At 20 and 21 DAS, PLA was 26.7% and 39.9% higher, respectively, in low N + Pk plants compared with low N controls. In contrast, Pk inoculation did not affect PLA under nitrogen‐sufficient conditions at any time point (Figure 1A).

FW responses emerged only at later stages. Significant effects of Pk inoculation under low nitrogen were detected at 21 DAS for both shoot and root FW (Figure 1B,D). At this time point, Pk inoculation increased shoot and root FW by 59.5% and 93.3%, respectively, relative to uninoculated low‐N controls. No FW effects of Pk were detected under high nitrogen conditions.

Dry weight (DW) responses mirrored these trends (Figure 1C,E). Nitrogen availability alone did not significantly affect DW at any time point. However, under low nitrogen, Pk inoculation led to a significant increase in both root and shoot DW at 21 DAS, corresponding to an approximately twofold increase compared with uninoculated low N plants (Figure 1C,E). No DW effects of Pk were observed under high N conditions.

Root system architecture was assessed destructively at 19, 20 and 21 DAS. Total root length increased significantly at 21 DAS in low N + Pk plants compared to low N, whereas no differences were observed at earlier time points. Partitioning the root system revealed distinct responses of primary seminal roots (PSRL) and lateral roots (LRL). PSRL was strongly influenced by nitrogen availability but was not affected by Pk inoculation at any time point (Figure 1F). In contrast, LRL was significantly increased in low N + Pk plants at 21 DAS compared to low N, with no corresponding effect under high N (Figure 1G). These results indicate that Pk selectively enhances lateral root development under nitrogen limitation.

Taken together, these data demonstrate that Pk enhances plant growth specifically under low N conditions. Increased root DW coincides with enhanced lateral root development, while increased shoot DW parallels the observed increase in leaf area. A consolidated summary of biomass and elemental responses at 21 DAS is provided in Supporting Information S7: Table 1.

### 3.2 Carbon and Nitrogen Content Responses to *Pseudomonas koreensis* Under Contrasting Nitrogen Regimes

**Figure 2:**
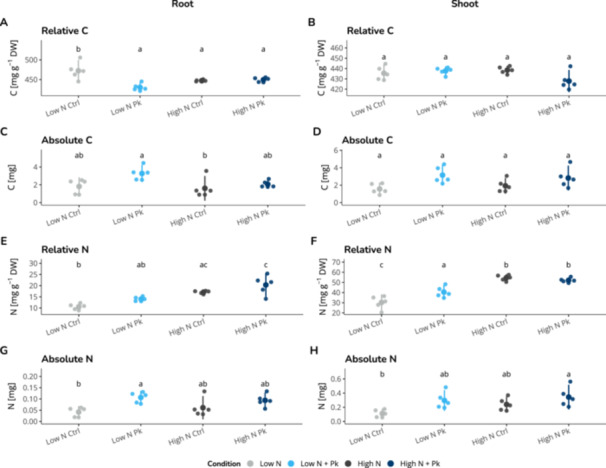
Carbon and nitrogen content of *Brachypodium distachyon* in response to nitrogen availability and *Pseudomonas koreensis*. Relative and absolute carbon and nitrogen contents were quantified at 21 days after sowing DAS in roots and shoots of plants grown under low‐ or high‐nitrogen conditions, with or without *P. koreensis* inoculation. (A and B) Relative carbon content [mg g^−1^ DW]; (C and D) absolute carbon content [mg per tissue]; (E and F) relative nitrogen content [mg g^−1^ DW]; (G and H) absolute nitrogen content [mg per tissue]. Relative values were converted from mg mg^−1^ DW to mg g^−1^ DW. Points represent individual biological replicates (*n* = 5); large symbols indicate group means ± 95% confidence intervals. Statistical comparisons were performed separately for each tissue at 21 DAS using two‐way ANOVA (Nitrogen × inoculation). Pairwise differences among the four treatment combinations were assessed using Tukey's HSD post hoc tests and are summarized as compact letter displays; groups sharing the same letter are not significantly different (*p* < 0.05).

At 21 DAS, relative carbon content (mg g^−1^ DW) did not differ between low‐ and high‐nitrogen control plants in either roots or shoots (Figure 2A,B). Under low nitrogen, inoculation with *Pseudomonas koreensis* (Pk) resulted in a significant reduction in relative root carbon content compared with uninoculated controls, whereas shoot carbon composition remained unaffected (Figure 2A,B). Absolute carbon content (µg per plant) did not differ significantly among treatments in either tissue at 21 DAS (Figure 2C,D).

Relative nitrogen content was strongly dependent on nitrogen supply in root and shoot, with high‐nitrogen control plants exhibiting higher nitrogen concentrations than low‐nitrogen controls in both tissues (Figure 2E). Pk inoculation under low nitrogen resulted in a significant increase in relative nitrogen content in shoots at 21 DAS, whereas no effect was observed under high‐nitrogen conditions (Figure 2E).

Absolute nitrogen content in the root revealed a significant effect of *Pk* under nitrogen limitation at 21 DAS. We also observed increased shoot nitrogen content in low N inoculated plants, reaching levels comparable to high‐nitrogen controls (Figure 2F–H). No effect of *Pk* on absolute nitrogen content was detected under high‐nitrogen conditions.

Even though Pk was able to grow on N‐deficient medium, a repetition of the experiment using ^15^N enriched medium, and measuring changes in the natural abundance of N isotopes in the plant did not provide conclusive evidence about biological N‐fixation by *Pk* in planta (Supporting Information S9: Supplemental Materials and Methods, Supplemental Results, Figures 6 and 9). Consequently, a time‐resolved multi‐omics approach was used to investigate the plant metabolic adaptation to the presence of the beneficial microbe.

### 3.3 Molecular Adaptations of Low N *Brachypodium* Plants to the Presence of Pk

To investigate molecular responses to *P. koreensis* (Pk) under contrasting nitrogen regimes, we performed a time‐resolved lipidomic analysis of *Brachypodium* roots at 19, 20 and 21 DAS (Harvest I; Supporting Information S9: Figure 1). Lipids were selected as a primary molecular readout given their central role in membrane structure, signalling and metabolic adaptation. At the final time point, proteomic profiling was conducted as a complementary data set to assess broader system‐level responses (Harvest II; Supporting Information S9: Figure 1).

### 3.4 The Lipidome of *Brachypodium* Mainly Responds to Different N Levels, With Very Few Pk‐Induced Lipid Changes

**Figure 3:**
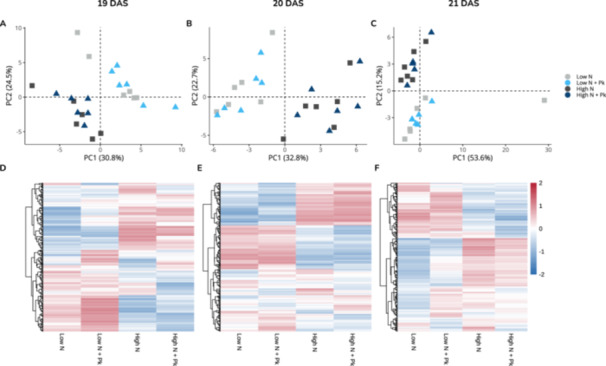
Time‐resolved lipidomic responses of *Brachypodium* roots under contrasting nitrogen regimes with and without *Pseudomonas koreensis*. Principal component analysis (PCA; A, B, C) and hierarchical clustering heatmaps (D, E, F) of root lipid profiles at 19, 20 and 21 DAS. PCA was performed on all manually curated lipid features (*n* = 255) following log_10_ transformation and Pareto scaling. Points represent individual biological replicates and are coloured by treatment condition. Heatmaps display mean *z*‐scored lipid abundances per condition (Low N, Low N + Pk, High N, High N + Pk), clustered by lipid species using Pearson's distance and average linkage. Nitrogen availability is the primary driver of lipidome separation across all time points, while *P. koreensis* inoculation induces only minor and transient changes. Panels correspond to 19 DAS (A and D), 20 DAS (B and E) and 21 DAS (C and F).

PCA of a curated subset of lipid features selected for annotation confidence and interpretability revealed a consistent separation between low‐ and high‐nitrogen conditions at all three time points (Figure 3A–C), indicating that nitrogen availability is the dominant driver of lipidome variation. In contrast, inoculation did not produce a clear separation under either nitrogen regime, consistent with a comparatively weak global effect on lipid composition.

Hierarchical clustering heatmaps supported this interpretation (Figure 3D–F). While individual lipid species showed changes in abundance following Pk inoculation, these effects were modest relative to nitrogen‐driven differences and lacked a consistent directional pattern across time points, suggesting the absence of coordinated lipidome remodelling.

To quantify Pk‐associated lipid changes, we performed per‐feature statistical testing within each time point using two‐way ANOVA followed by Tukey's HSD post hoc tests. This analysis identified a limited number of lipid features exhibiting significant abundance differences between inoculated and uninoculated plants, spanning multiple lipid classes, including lysophospholipids (LPE), phosphatidylethanolamines (PE), diacylglycerols (DG), phosphatidylglycerols (PG) and several unannotated features (Supporting Information S4: Table 6). No lipid class displayed coordinated regulation across time points.

Pk‐associated lipid responses were most pronounced at 19 DAS, where the majority of significant differences were observed, primarily under low‐nitrogen conditions. In contrast, substantially fewer lipid features differed at 20 DAS, and no significant Pk‐associated changes were detected at 21 DAS. This temporal pattern indicates that lipidomic responses to Pk inoculation are transient rather than sustained. Time‐resolved abundance changes of significantly Pk‐responsive lipid features are shown in Supporting Information S9: Figure 7.

Collectively, these results demonstrate that lipidomic variation in *Brachypodium* roots is overwhelmingly structured by nitrogen availability, with Pk inoculation eliciting only weak, temporally restricted effects on individual lipid species.

### 3.5 The *Brachypodium* Proteome Shows Enhanced Responsiveness to Pk Inoculation Under Nitrogen Limitation

Proteomic analyses were conducted for two purposes: (i) to confirm successful plant inoculation by detecting Pk‐derived proteins as a qualitative control, and (ii) to assess how *Brachypodium* protein abundances respond to Pk inoculation under contrasting nitrogen conditions.

#### 3.5.1 Detection of Pk‐Derived Proteins Confirms Inoculation

A qualitative search against a non‐redundant *P. koreensis* protein database was used to identify bacterial proteins present in plant extracts (Harvest II; Supporting Information S9: Figures 1 and 8). In inoculated samples, 30 Pk‐specific proteins and 4 proteins with sequences conserved across multiple bacterial species were detected. In contrast, uninoculated samples contained no Pk‐specific proteins in most cases. A small number of control samples contained low‐abundance Pk‐specific peptides; however, their frequency and abundance were markedly lower than in inoculated samples, confirming successful inoculation and minimal cross‐contamination.

#### 3.5.2 Global Structure of the *Brachypodium* Proteome

**Figure 4:**
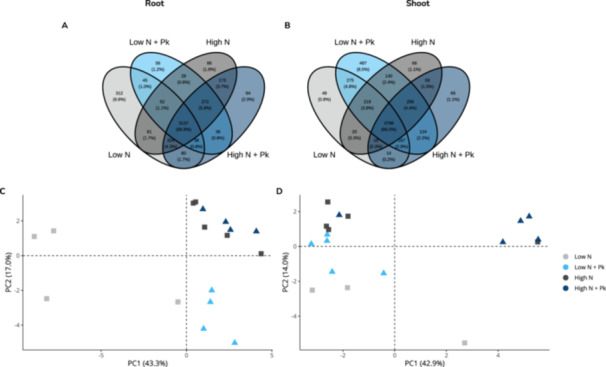
Proteomics data overview. Venn diagrams of proteins with 100% valid values detected in at least one condition in roots (A) and shoots (B). PCA of root (C) and shoot (D) proteomes based on proteins with complete data across all conditions.

In total, 5660 proteins were identified in roots and 6656 proteins in shoots. Of these, 3135 root proteins and 3975 shoot proteins were consistently quantified with 100% valid values within at least one experimental condition (Figure 4A,B). The numbers of differentially abundant proteins detected in each comparison are summarized in Supporting Information S1: Table 2.

Across tissues, nitrogen availability remained the dominant source of proteome‐wide variation at 21 DAS (16 days after inoculation). PCA performed on proteins quantified across all conditions revealed a clear separation driven by nitrogen status in both roots and shoots (Figure 4C,D). However, nitrogen limitation markedly enhanced the proteomic response to Pk inoculation.

Consistent with this observation, volcano plot analyses showed that Pk inoculation under low nitrogen resulted in substantially more significant protein abundance changes than under high‐nitrogen conditions (Supporting Information S9: Figure 10). Based on this strong nitrogen dependency, subsequent analyses focus primarily on Pk‐associated responses under low nitrogen.

#### 3.5.3 Functional Context of Nitrogen‐ and Pk‐Associated Proteomic Changes

To contextualize these responses, enriched Gene Ontology (GO) biological process terms associated with nitrogen availability (High N control vs. Low N control) were compared to those associated with Pk inoculation under low nitrogen (Low N + Pk vs. Low N control) in both roots and shoots (Supporting Information S9: Figure 11). In roots, both contrasts were associated with broad shifts across multiple biological process categories, with partially distinct patterns between nitrogen‐driven and Pk‐associated responses (Supporting Information S9: Figure 11A). In shoots, fewer GO terms were enriched overall, and the dominant functional categories differed between nitrogen and Pk contrasts (Supporting Information S9: Figure 11B).

To obtain a system‐level view of plant adaptation to *P. koreensis* under nitrogen limitation, we next focused on protein groups implicated in lipid metabolism (to complement the lipidomic analysis) and nitrogen metabolism and transport.

### 3.6 Weighted Gene Co‐Expression Network Analysis Reveals Nitrogen‐Structured Proteomic Organization With Conditional Modulation by *Pseudomonas*

To assess coordinated proteomic responses beyond individual protein changes, we performed WGCNA on the root proteomics data set. This approach organized quantified proteins into co‐expression modules based on shared abundance patterns across treatments, enabling evaluation of system‐level responses to nitrogen availability and *Pseudomonas* inoculation. Importantly, modules associated with *P. koreensis* inoculation were detected only under low‐nitrogen conditions and were also significantly associated with nitrogen availability.

**Figure 5:**
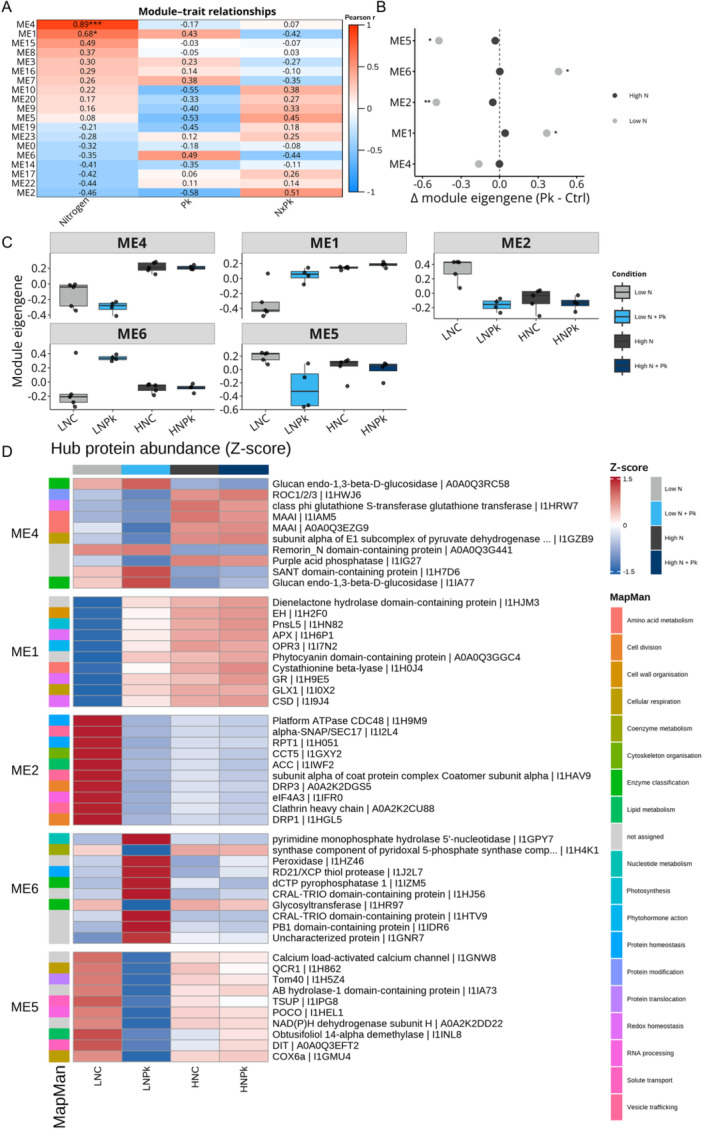
Nitrogen status defines system‐level proteome modules, with conditional modulation by *Pseudomonas*. (A) Module–trait relationships (Pearson's *r*) between module eigengenes and trait vectors (Nitrogen, Pk, NxPk). Asterisks denote significance after Benjamini–Hochberg correction across modules within each trait (*FDR < 0.05; **FDR < 0.01; ***FDR < 0.001). (B) Effect sizes of Pk on module eigengenes within nitrogen backgrounds (Δ eigengene = Pk − Ctrl), estimated from linear models and simple‐effect contrasts (Yes vs. No) within each nitrogen level (emmeans). Asterisks denote Benjamini–Hochberg–adjusted significance across modules within each nitrogen stratum (*FDR < 0.05; **FDR < 0.01; ***FDR < 0.001). (C) Distribution of module eigengene values across treatments. Boxes show medians and interquartile ranges; whiskers denote 1.5× IQR; points represent individual samples. (D) Heatmap of scaled mean protein abundances (*z*‐scores) for hub proteins from focal modules across treatments. Proteins are ordered by module and intramodular connectivity. Side annotations indicate MapMan functional categories and module identity.

Module–trait correlation analysis identified nitrogen availability as the dominant factor structuring the root proteome (Figure 5A). Complete module–trait correlation statistics are provided in Supporting Information S9: Table 3. Several large modules showed strong positive or negative correlations with nitrogen status, whereas no module exhibited a strong or exclusive association with *Pseudomonas* treatment alone. Instead, *Pseudomonas*‐associated effects were detected primarily within nitrogen‐associated modules and were most pronounced under nitrogen‐limiting conditions, consistent with conditional modulation of an existing nitrogen‐defined proteomic framework rather than establishment of a microbe‐specific programme.

Analysis of module eigengenes supported this interpretation. For multiple nitrogen‐associated modules, eigengene values under low nitrogen + *Pseudomonas* shifted in the direction of those observed under high‐nitrogen control conditions, whereas *Pseudomonas* inoculation under high nitrogen resulted in comparatively minor changes (Figure 5B,C). This pattern indicates that *Pseudomonas* inoculation alters the position of low‐nitrogen plants within a nitrogen‐structured proteomic landscape, rather than generating a distinct proteomic state.

To further characterize the biological composition of these responses, we examined hub proteins within selected nitrogen‐responsive modules. Hub proteins are used here as descriptive markers of coordinated module behaviour rather than as evidence of direct regulatory control. Hub proteins were defined as the top‐ranked proteins by module membership (kME), representing highly connected and representative components of each module. Full module membership assignments and intramodular connectivity values (kME) for all quantified proteins are provided in Supporting Information S3: Table 5. Across modules, hub proteins displayed diverse functional annotations, with MapMan bin categories dominated by unassigned proteins (bin 35), protein metabolism (bin 19), enzyme classes (bin 50) and amino acid‐related processes (bin 4) (Figure 5D). No single functional category uniquely defined any module, indicating that nitrogen availability coordinates broad metabolic and cellular processes rather than isolated pathways. Unassigned proteins (MapMan bin 35) and broadly classified enzyme categories were retained to avoid annotation bias; complete MapMan bin distributions for all modules are provided in Supporting Information S2: Table 4.

Consistent with this interpretation, proteins within individual modules showed coordinated abundance shifts across treatments, indicating module‐level responses rather than uniform regulation of individual proteins. Together, these patterns reinforce the conclusion that nitrogen availability imposes a system‐level organization of the root proteome. Within this framework, *Pseudomonas* inoculation under nitrogen limitation induces structured, module‐internal adjustments.

A parallel WGCNA performed on the shoot proteome revealed a similar but attenuated organization, with nitrogen again emerging as the primary determinant of module structure and comparatively weaker *Pseudomonas*‐associated effects (Supporting Information S9: Figure 12). This supports a predominantly root‐centred proteomic response to nitrogen availability and microbial inoculation.

*Pseudomonas* inoculation under low nitrogen conditionally modulates this organization, shifting eigengene values towards those observed under high‐nitrogen conditions without establishing distinct microbe‐specific modules.

### 3.7 From Network‐Level Organization to Pathway‐Level Responses

The WGCNA revealed that nitrogen availability imposes a dominant, system‐level organization on the root proteome, with *Pseudomonas* inoculation exerting conditional effects within this nitrogen‐defined framework. Rather than forming microbe‐specific co‐expression modules, *Pseudomonas*‐associated responses were embedded within nitrogen‐responsive modules and were most pronounced under nitrogen limitation. This organization provides a basis for identifying biological processes that consistently reflect the nitrogen‐structured proteomic state and its modulation by bacterial inoculation. Importantly, no co‐expression module was uniquely associated with *Pseudomonas* inoculation independent of nitrogen status.

To translate these network‐level patterns into interpretable biological responses, we examined pathway‐level protein abundance changes guided by module composition, hub protein annotations, and link them to the independent elemental and lipidomic measurements. We focused on lipid metabolism as a representative downstream metabolic process, and on nitrogen and amino‐acid metabolism as the core functional axis underlying the nitrogen‐associated proteomic reprogramming. These analyses do not imply direct regulatory roles for individual hub proteins but rather hub proteins serve as representative markers of coordinated responses within nitrogen‐associated modules. This network structure provides a rationale for examining pathway‐level responses represented within nitrogen‐associated modules.

### 3.8 Lipid Metabolism Proteins Correspond to Pathways Represented in the Lipidomic Data Set

**Figure 6:**
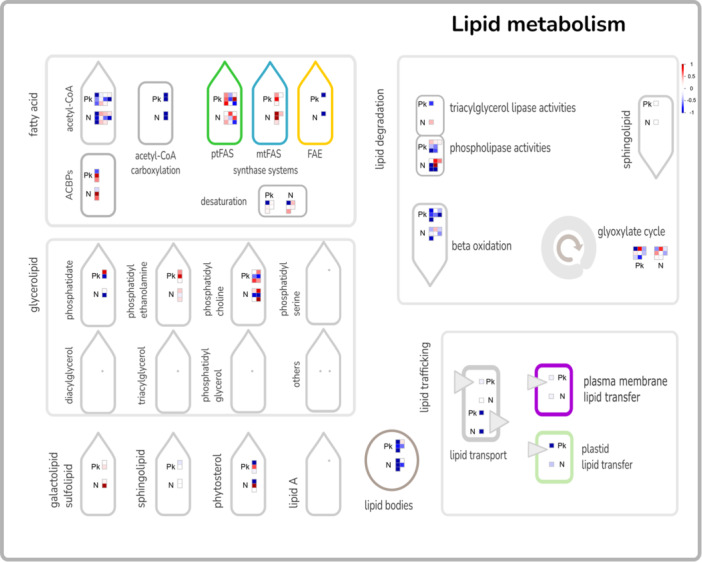
Lipid metabolism is coordinated by nitrogen availability and modulated by Pk in roots. Heatmap showing log_2_ fold changes in the abundance of root proteins involved in lipid metabolism, restricted to proteins quantified across 100% of samples (no imputation). Colours indicate relative changes (blue, higher abundance; red, lower abundance) compared with the indicated reference condition. Comparisons shown are High N control versus Low N control (nitrogen effect‐N) and Low N + *Pk* versus Low N control (Pk effect‐Pk). Proteins were mapped using MapMan v3.5.1R2 (Thimm et al. 2004) with a custom pathway derived from lipid metabolism (X4.5, R5.0); mapping files were generated using Mercator4 (Schwacke et al. 2019). Mapped proteins, log_2_ fold changes and statistical significance are provided in Supporting Information S1: Table 2. ptFAS, plastidial fatty acid synthase; mtFAS, mitochondrial fatty acid synthase; FAE, fatty acid elongase.

Given the nitrogen‐dependent organization of the proteome revealed by WGCNA, we examined proteins associated with lipid metabolism in roots. These proteins mapped to pathways involved in fatty‐acid synthesis, fatty‐acid elongation (FAE), membrane phospholipid metabolism, lipid degradation and lipid transfer processes (Figure 6).

Nitrogen availability strongly influenced the abundance of several proteins associated with fatty‐acid synthesis and membrane lipid metabolism. In roots, comparisons between high‐ and low‐nitrogen controls showed coordinated abundance differences among proteins involved in plastidial and mitochondrial fatty‐acid synthesis (ptFAS, mtFAS), FAE and phospholipid metabolism (Figure 6).

Under low nitrogen, *P. koreensis* inoculation was associated with changes in the abundance of a subset of these lipid‐metabolism proteins relative to low‐nitrogen controls. For several proteins, the direction of abundance change observed in low N + Pk plants resembled that observed between high‐ and low‐nitrogen control plants. In contrast, under high nitrogen conditions, only minor differences were observed between Pk‐inoculated and uninoculated plants.

Together, these results show that proteins associated with lipid metabolism display nitrogen‐dependent abundance patterns, with additional Pk‐associated differences primarily observed under low nitrogen.

### 3.9 Nitrogen and Amino Acid Metabolism Reflect Nitrogen‐Structured Network States and *Pseudomonas*‐Associated Modulation at Low Nitrogen

Given the nitrogen‐dependent organization of the proteome and the increased plant nitrogen content observed under *Pseudomonas* inoculation, we examined proteins involved in nitrogen uptake and central nitrogen and amino‐acid metabolism. These pathways represent the primary interface between inorganic nitrogen availability and plant growth and therefore provide a functional readout of the nitrogen‐structured proteomic state.

**Figure 7:**
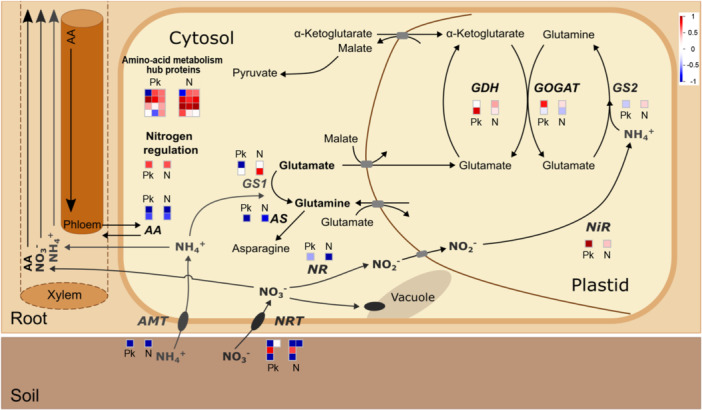
Nitrogen‐responsive hub proteins highlight coordinated regulation of central amino‐acid metabolism in roots. Schematic representation of central nitrogen and amino‐acid metabolism in roots, annotated with WGCNA‐identified hub proteins quantified across all samples. Coloured squares indicate log_2_ fold changes relative to low‐nitrogen control (LNC), shown as Low N + Pk – LNC (Pk effect) and High N control – LNC (nitrogen effect). Only root proteins meeting the 100% data‐completeness criterion are shown. Pathway structure is based on MapMan (version 3.5.1R2; Thimm et al. 2004) nitrogen uptake and assimilation modules with custom modifications; mapping files were generated using Mercator4 (Schwacke et al. 2019). Mapped proteins, log_2_ fold changes and statistical significance are provided in Supporting Information S1: Table 2. AA, amino acids; AMT, ammonium transporter; AS, asparagine synthase; GDH, glutamate dehydrogenase; GOGAT, glutamine oxoglutarate aminotransferase; GS1, cytosolic glutamine synthetase; GS2, plastidial glutamine synthetase; NiR, nitrite reductase; NR, nitrate reductase; NRT, nitrate transporter.

As expected, nitrogen availability strongly influenced the abundance of nitrate and ammonium transporters and assimilation enzymes. In roots, high N control plants showed reduced abundance of several nitrate and ammonium transporters (NRT, AMT, respectively) compared with low N controls, consistent with canonical nitrogen sufficiency responses (Figure 7). Components of the glutamine–glutamate cycle exhibited isoform‐specific regulation, indicating redistribution of assimilation capacity rather than uniform up‐ or down‐regulation.

Under low nitrogen, *Pseudomonas* inoculation was associated with a reconfiguration of nitrogen metabolism rather than a uniform increase in protein abundance across the pathway. In low N inoculated roots, most nitrate and ammonium transporters were unchanged or reduced in abundance relative to low N controls, while one MFS domain–containing transporter (I1H1D1_bradi) increased in abundance. Nitrate reductase abundance decreased whereas nitrite reductase increased, and the nitrogen regulatory P‐II protein accumulated strongly, consistent with altered nitrogen sensing and allocation. Enzymes of the glutamine–glutamate cycle showed opposing changes among isoforms, indicating redistribution within existing assimilation pathways (Figure 7).

In shoots, low N + Pk plants displayed a coordinated increase in proteins associated with nitrate and ammonium assimilation and amino‐acid biosynthesis, including glutamine synthetase and asparagine synthase (Supporting Information S9: Figure 13). This pattern is consistent with enhanced nitrogen assimilation and redistribution to above‐ground tissues.

Under high nitrogen, *Pseudomonas* inoculation elicited only minor changes in nitrogen metabolism in both roots and shoots, paralleling the weak phenotypic and proteomic responses observed under high nitrogen conditions.

Together, these results indicate that nitrogen and amino‐acid metabolism represent the primary biological axis through which the nitrogen‐structured proteomic network is expressed. Under low nitrogen, Pk inoculation was associated with protein abundance patterns that more closely resembled those observed under high‐nitrogen control conditions.

## 4 Discussion

Our results demonstrate that *P. koreensis* promotes plant growth specifically under nitrogen‐limiting conditions, leading to increased biomass and higher total plant nitrogen content after 3 weeks of growth (Figures 1, 2 and 2). Under low nitrogen, inoculated plants produced nearly twice the biomass of uninoculated controls and accumulated more nitrogen in shoots, whereas no growth or nitrogen effects were observed under high nitrogen conditions (Figures 1B–E and 2E–H). Relative nitrogen concentration in shoots increased under low N + Pk compared with low N controls, indicating that enhanced nitrogen accumulation reflects a combination of increased biomass and subtle shifts in tissue nitrogen levels (Figure 2E–H). In parallel, relative root carbon content was reduced in inoculated plants without changes in absolute carbon content (Figure 2A–D), consistent with altered carbon allocation rather than increased carbon uptake. Together, these elemental patterns indicate a redistribution of resources within the plant and improved nitrogen‐use efficiency under nitrogen limitation, consistent with coordinated reallocation and assimilation (Xu et al. 2012; Liu et al. 2022). Compared with Kuang et al. (2022), our study extends previous findings by integrating lipidomics and proteomics at the whole‐plant level, using a *Pseudomonas* strain with putative nitrogen‐fixing capacity, and resolving the temporal dynamics of growth promotion under contrasting nitrogen regimes.

Root protein abundance patterns in low N + Pk plants shifted towards those observed under high‐nitrogen conditions, and this convergence was evident at the level of co‐expression modules and pathway‐associated proteins (Figures 5, 6, 7).

Conceptually, these findings distinguish between microbial traits and host physiological state as determinants of growth promotion under nutrient stress. This perspective shifts the focus from microbe‐centric explanations towards host‐integrated responses that emerge only under specific nutritional contexts (Trivedi et al. 2020; Kawa and Brady 2022). Such context dependency may explain why reproducible growth‐promotion effects of PGPB are often difficult to detect under nutrient‐replete conditions (Backer et al. 2018; Compant et al. 2019; Sessitsch et al. 2019).

Proteomic analyses support this interpretation by showing that nitrogen availability remains the dominant factor structuring the plant proteome, while *Pseudomonas* inoculation under low nitrogen induces conditional modulation within this nitrogen‐defined framework (Figures 4 and 5).

### 4.1 Nutrients and Bacteria Influence Plant Phenotype and Physiology on Different Temporal Scales

In this study, differences in leaf area due to nitrogen supply were already quantifiable early, at 5 DAS, consistent with the rapid plant response to mineral nitrogen availability. By contrast, the influence of the plant growth–promoting bacterium *Pseudomonas koreensis* (Pk) became consistently visible only at 19 DAS, that is, 14 days after inoculation. This timing is slightly faster than reported in a related study using *Brachypodium* grown under two nitrogen conditions in a gnotobiotic microchamber system with liquid medium and inoculated with *H. seropedicae*, where increased leaf area due to bacterial inoculation was also detected 19 days after inoculation (Kuang et al. 2022).

At the root level, both *Herbaspirillum* and *Pseudomonas* were found to increase root length under low nitrogen; however, while *Herbaspirillum* primarily enhanced primary root length (Kuang et al. 2022), *Pseudomonas* increased total root length predominantly through stimulation of lateral root development (Figure 1F,G). This comparison suggests that both the identity of the bacterial inoculant and the environmental context shape the timing and mode of plant responses (Bashan and de‐Bashan 2010; Kuang et al. 2022; Kawa and Brady 2022; Ke et al. 2025; Xiaoming et al. 2025) but also highlights that these observed traits represent only a subset of the complexity inherent to plant–microbe interactions.

In natural settings, such interactions are further influenced by dynamic root development programmes (Kawa and Brady 2022) and by shifts in microbiome composition across root‐associated compartments, ranging from the endosphere to the rhizosphere and bulk soil (Singer et al. 2021; Bai et al. 2022). Given the diversity of microbes encountered by plant roots, a wide range of microbe‐associated molecular patterns and damage‐associated molecular patterns contribute to the regulation of these interactions and to the integration of microbial cues with plant developmental and nutritional signals (Zhou et al. 2020; Ma et al. 2021; Kawa and Brady 2022).

### 4.2 Multiple Mechanisms of *Pseudomonas koreensis*–Mediated Growth Promotion and Remaining Open Questions

The increased growth and nitrogen content observed in Pk–inoculated plants under low nitrogen likely arise from multiple, partially overlapping mechanisms rather than a single dominant process. Although Pk harbours genes associated with nitrogen fixation, has confirmed nitrogenase activity in vitro (Rafikova et al. 2016), and is capable of growth on nitrogen‐deficient media, this is not sufficient evidence for atmospheric nitrogen fixation in planta (Dixon and Kahn 2004; Santi et al. 2013). The δ^15^N data obtained in this study did not reveal a clear isotopic signature indicative of substantial in‐planta biological nitrogen fixation, necessitating a cautious interpretation of bacterial contributions to plant nitrogen nutrition.

An alternative, and not mutually exclusive, explanation for the increased nitrogen content of inoculated plants is enhanced nitrogen acquisition or utilization efficiency driven by plant physiological responses. Under low nitrogen, Pk–inoculated plants exhibited increased LRL at later developmental stages, a trait commonly associated with improved soil exploration and nutrient uptake under nitrogen limitation (Gruber et al. 2013; Giehl and von Wiren 2014; Y. Wang et al. 2023). However, because increases in LRL were detected after increases in plant nitrogen content were already apparent, changes in root architecture alone cannot fully explain the observed nitrogen accumulation. This temporal offset suggests that additional mechanisms, such as altered transporter activity, nitrogen assimilation efficiency, or internal nitrogen redistribution, contribute to the phenotype (Masclaux‐Daubresse et al. 2010; Krapp 2015).

Consistent with this interpretation, proteomic analyses revealed adjustments in the abundance of nitrogen transporters, assimilation enzymes and regulatory proteins under low N + Pk conditions. These changes did not reflect uniform upregulation of nitrogen uptake pathways but rather a reconfiguration of nitrogen‐related processes, including isoform‐specific regulation of transporters and enzymes involved in the glutamine–glutamate cycle (Canales et al. 2014; Vidal et al. 2020). Such patterns are consistent with modulation of nitrogen sensing and allocation rather than activation of a distinct microbial nitrogen acquisition pathway (Masclaux‐Daubresse et al. 2010; Feng et al. 2020).

Together, these observations support a model in which *P. koreensis* promotes plant growth under nitrogen limitation through coordinated modulation of plant nitrogen physiology, resource allocation and root system development. While bacterial nitrogen fixation cannot be excluded entirely, the available evidence favours a scenario in which plant‐encoded responses dominate the observed phenotype. Future work employing isotopic labelling under controlled conditions, bacterial mutants impaired in nitrogen metabolism, or plant genotypes altered in nitrogen uptake or signalling pathways will be required to disentangle the relative contributions of these mechanisms (Glick 2012; Santi et al. 2013).

Additionally, the spatial and temporal heterogeneity of bacterial activity at the root surface or within plant tissues remains unresolved and should be addressed in future studies. Finally, while proteomic and lipidomic patterns indicate coordinated host responses, they do not establish causal relationships between specific molecular changes and growth outcomes. These limitations motivate future work combining genetic perturbations and targeted isotope tracing to directly test causal links.

### 4.3 Lipid and Energy Metabolism Adjustments Accompany Nitrogen‐Dependent Growth Responses

Nitrogen availability is closely linked to plant lipid metabolism, as carbon allocation, membrane composition and energy balance must be coordinated with nitrogen status to support growth. In this study, lipidomic and proteomic analyses revealed modest but consistent changes in lipid composition and lipid‐associated proteins under low nitrogen, with additional modulation following *P. koreensis* inoculation. These changes were most pronounced at early and intermediate time points and were attenuated under high nitrogen conditions, indicating that lipid metabolism responds primarily to nitrogen status rather than primarily to bacterial presence (Masclaux‐Daubresse et al. 2010; Okazaki and Saito 2014; Schillaci et al. 2021). Given the modest magnitude, temporal restriction and lack of coordinated lipid‐class–level regulation, lipid remodelling is unlikely to represent a primary driver of the growth phenotype, but rather a secondary adjustment within a nitrogen‐structured physiological state.

Under low nitrogen, *P. koreensis* inoculation was associated with alterations in membrane lipid classes and reduced abundance of proteins involved in lipid degradation at later stages, consistent with shifts in membrane turnover and cellular energy economy during nitrogen stress (Gaude et al. 2007; Okazaki and Saito 2014). In this context, lipid metabolism likely contributes to maintaining membrane integrity and energetic balance under nitrogen limitation rather than directly promoting biomass accumulation.

## 5 Concluding Remarks

In summary, growth promotion by *P. koreensis* in *B. distachyon* was conditional on nitrogen limitation and was associated with coordinated physiological and proteomic adjustments rather than the establishment of a distinct microbial nitrogen acquisition pathway. Under low nitrogen, inoculated plants exhibited increased biomass and nitrogen accumulation accompanied by proteomic patterns that shifted towards those observed under high nitrogen conditions. These responses were evident at the level of co‐expression networks, pathway‐associated proteins and metabolic readouts, indicating a system‐level adjustment of plant nitrogen physiology rather than isolated enzyme‐specific effects (Langfelder and Horvath 2008; Masclaux‐Daubresse et al. 2010). Importantly, the absence of a robust δ^15^N signature argues against substantial in‐planta nitrogen fixation under the conditions tested, underscoring the need for cautious interpretation of bacterial contributions to plant nitrogen nutrition (Dixon and Kahn 2004; Santi et al. 2013).

The data instead support a model in which *P. koreensis* enhances plant performance under nitrogen stress by modulating plant‐encoded processes related to nitrogen sensing, assimilation, allocation and root system architecture. Lipid and energy metabolism emerge as integrated components of this response, likely facilitating metabolic flexibility and cellular stability during prolonged nitrogen limitation rather than directly driving growth promotion (Masclaux‐Daubresse et al. 2010; Okazaki and Saito 2014). Together, these findings reinforce the view that PGPB primarily act by reshaping host physiological states in a context‐dependent manner.

More broadly, this work highlights the value of network‐based and time‐resolved approaches for disentangling complex plant–microbe interactions under nutrient stress. By explicitly distinguishing descriptive system‐level responses from untested mechanistic hypotheses, this study provides a framework for future work aimed at resolving causal links, for example, using bacterial mutants, plant nitrogen signalling mutants or targeted isotopic tracing under controlled conditions (Langfelder and Horvath 2008; Glick 2012). Such approaches will be essential for translating descriptive multi‐omics patterns into predictive understanding of plant–microbe interactions in agricultural and natural systems.

## Conflicts of Interest

The authors declare no conflicts of interest.

## Supporting information

Supporting File 1

Supporting File 2

Supporting File 3

Supporting File 4

Supporting File 5

Supporting File 6

Supporting File 7

Supporting File 8

Supporting File 9

## Data Availability

The mass spectrometry proteomics data have been deposited to the PRIDE repository (Perez‐Riverol et al. 2025) with the with the dataset identifier PXD050676. The metabolomics data have been deposited to MetaboLights (Yurekten et al. 2024) repository with the study identifier MTBLS12763 (https://www.ebi.ac.uk/metabolights/MTBLS12763).
