# Supplementary material for: Systems‐Level Plant Responses Reveal Pseudomonas‐Mediated Growth Promotion in Brachypodium Under Nitrogen Limitation
Source: Plant Cell Environ. 2026 May 25;49(9):6396–412. doi: 10.1111/pce.70615 (PMC13436448; doi:10.1111/pce.70615)
Supplement: Supplementary file 1 [file PCE-49-6396-s005.pdf]

Supplemental Material to the manuscript:

Systems-level Plant Responses Reveal Pseudomonas-Mediated Growth Promotion in Brachypodium Under Nitrogen Limitation

Sanow, S., Mantz, M., Wissel, H., Bhatnagar, A., E. Sturm, J. M. Kelm, Walker, R., Lücke, A., Schaaf, G., Huesgen, P., Roessner, U., Watt, M. and Arsova, B.

This document contains:

1. Supplemental Materials and Methods – page 2
2. Supplemental Results – page 3
3. Supplemental Figures – page 6
4. Supplemental Tables – page 19

1. Supplemental Materials and Methods

^{15}N natural abundance and enrichment analysis

An independent experiment was conducted to assess plant nitrogen isotope composition. Sixty plants were grown under either natural abundance (non-enriched) or ^{15}N-enriched conditions. For each enrichment regime, 30 plants were distributed across six treatments (n = 5 per treatment): nitrogen-depleted control (Zero N), Zero N + Pk, nitrogen-limited control (Limited N), Limited N + Pk, nitrogen-sufficient control (Sufficient N), and Sufficient N + Pk. Growth conditions and inoculation procedures were as described above, except that enriched treatments received 300 ‰ double-labelled $^{15}NH_4{}^{15}NO_3$ (Campro Scientific; CAS 43086-60-8). Plants were harvested at 21 days after sowing (DAS), flash-frozen, and lyophilized.

Dried tissue was homogenized by cutting into small fragments. Approximately 0.5–1.5 mg dry material was weighed into tin capsules for stable isotope analysis. Samples were combusted at 1060 °C in an elemental analyser (Flash 2000, Thermo Fisher Scientific) coupled to an isotope ratio mass spectrometer (Delta V Plus, Thermo Fisher Scientific). International and laboratory standards (HZM sediment, acetanilide, alanine, histidine, serine) were measured alongside samples. Calibration and scale normalization were performed using IAEA-N-2 ($\delta^{15}N$ = 20.3 ‰), IAEA-N-1 (0.4 ‰), and USGS25 (–30.4 ‰).

Isotope ratios are reported in δ notation (‰) relative to atmospheric N_2 (AIR) according to:

$\delta^{15}N = (R\_sample / R\_standard - 1)$,

where R denotes the $^{15}N/^{14}N$ ratio. Analytical precision, based on replicate standard measurements, was < 0.2 ‰ (Coplen, 2011).

Growth of bacteria on nitrogen-deficient media

N-fixation ability was tested using nitrogen-deficient malate medium (NDM; Weselowski et al., 2016). *Pseudomonas koreensis* Ps 9-14^{T}, *P. syringae* pv lapsa, *P. taiwanensis* VLB120, and *E. coli* DH5α were pre-cultured in LB (28 °C, 220 rpm, overnight). Bacteria were spread on LB and nitrogen-deficient malate medium (NDM) supplemented with 50 mg/L yeast extract (NDM+) plates. After one day of incubation, colonies were spread on LB and NDM plates, followed by incubation for 3 days at 28 °C and 36 °C for *Pseudomonas* and *E. coli*, respectively.

2. Supplemental Results

Pseudomonas koreensis can grow on Nitrogen deficient medium *in vitro* but changes to the $^{15}N/^{14}N$ isotopic distribution of inoculated plants were not significant.

To investigate the N-fixing hypothesis, we submitted Pk to zero N conditions (no N source was supplemented in the media) in an *in vitro* plate assay. Pk was able to grow on N-defficient medium, while an *E. coli* control could not. In addition, we saw that *P. syringae* and *P. taiwanensis* could also form colonies on N-free medium (Supplemental Figure S9).

Accordingly, we expected that plants grown together with N-fixing organisms under N-limiting conditions would show a decreased δ15N signature vs. air compared to control plants grown without N-fixing organisms (Unkovich, 2013) due to the low d15N signature of biologically fixed N.

Measuring the δ15N signature of *Brachypodium* (Supplemental methods), after inoculation with Pk, under three N availability regimes revealed a trend to decreased δ15N in the zero N condition in roots (29.1 %) and shoots (12.8 %) and in low N conditions in both root (27.3 %) and shoot tissues (11.1 %) compared to their uninoculated control (Supplemental Fig. 6 a, b). Under sufficient N, only shoots showed a trend of decreased δ15N signature (Supplemental Fig. 6 a, b). It seems plausible to assume that under these conditions, N fixation does not occur to any significant extent, and that the response in shoots was due to enhanced biological discrimination against 15N. However, the variation among our biological replicates was very large, leading to inconclusive results for in planta N-fixation. Studies with a higher gradation of N conditions could potentially provide further insights.

Kinase families in *Brachypodium* respond to N availability and Pk inoculation

We compared the abundance of the various kinase families (Supplemental Fig. 12, Supplemental Table 1), including many that have an extracellular domain and function across the plasma membrane. Here, we highlight the Ca^{2+}/calmodulin-dependent protein kinase (CAMK) group, with particular focus on the SnRK1 kinase complex, a regulatory hotspot for energy homeostasis and stress responses including nutrient signaling (Broeckx et al., 2016; Crepin & Rolland, 2019).

In roots, the SnRK2 SNF1-related protein kinase SAPK6 (I1IAE4_BRADI) was more abundant in high N Controls compared to low N Controls (1.05 log2FC), which was also observed after inoculation of low N plants with Pk (0.54 log_2FC) and mildly after inoculation of high N plants (0.29

log_2FC). The regulatory subunit beta of SNF1-related SnRK1 kinase complex (I1HI64_BRADI) accumulated under high N controls vs. low N controls (0.83 log_2FC), whereas SAPK3 (I1I692_BRADI), SAPK7 (I1IXT7_BRADI) and SAPK8 (I1GMZ0_BRADI) were generally reduced compared to low N controls, with only slight reductions in high N + Pk. In contrast, the regulatory subunit betagamma of SNF1-related SnRK1 kinase complex (I1GKM6_BRADI) decreased strongly under high N controls compared to low N controls (-0.73 log_2FC) and after inoculation of low N plants (-0.91 log_2FC). However, the regulatory subunit gamma of SNF1-related SNRK1 kinase complex (I1HNY1_BRADI), remained unchanged in high N controls compared to low N controls (0.02 log_2FC), but increased moderately in low N inoculated plants compared to low N control (0.56 log_2FC). It was also moderately less abundant after inoculation of high N plants (-0.43 log_2FC) compared to high N controls.

The biggest differences were observed in CDPK protein kinases. Expression of two CDPKs (I1GMG8_BRADI; I1H269_BRADI), decreased under high N controls vs. low N controls (-0.39; -0.18 log_2FC, respectively) but increased after low N + Pk inoculation (0.25; 0.37 log_2FC), with little change under high N + Pk. Another CDPK (I1J0Z2_BRADI) and the CRK kinase (I1GX02_BRADI) were strongly reduced under high N versus low N (–1.16; –0.9 log_2FC) and low N + Pk (–0.75; –1.11 log_2FC), but remained unchanged under high N + Pk.

As part of the CMGC kinase superfamily, the MAP kinase I1I5N8_BRADI was found to be moderately less abundant in the high N Controls compared to low N controls (-0.51 log_2FC), much less abundant after inoculation of low N plants (-1.33 log_2FC) and unchanged after inoculation of high N plants.

Overall, in roots the abundance ratios of many kinases under high N versus low N were mirrored in low N + Pk versus low N, suggesting overlapping regulation. Exceptions from this trend are considered potential candidates for adaptation to beneficial microbes.

In the shoot, on the other hand first we see fewer significantly regulated kinases and second more of them have opposite behaviour between the high N control vs low N control, and low N + Pk vs low N control. This is in line with our hypothesis that they might be involved in the response to PGPB.

In the case of L-type lectin receptor kinases which are implicated in plant immunity (Wang & Bouwmeester, 2017), we see higher abundance in high N than in low N, but at low N + Pk decreases the abundance of kinase A0A0Q3KPD2_BRADI.

An opposite behaviour was seen in a representative of the CMGC kinase superfamily (comprising of CDK, MAPK, GSK3 and CLK families), I1I8I8_BRADI, the CAMK kinase superfamily proteins (I1H626_BRADI, I1HPI9_BRADI and I1GKM6_BRADI) and the LRR kinases (I1ID77_BRADI, A0A0Q3EYZ3_BRADI and A0A2K2D9S6_BRADI). These kinases are downregulated under high nitrogen compared to deficiency, but upregulated at low N in the presence of Pk. We speculate that these kinases (and other regulated in a similar manner) represent internal plant regulatory adaptations to presence of the beneficial microbe.

3. Supplemental figures

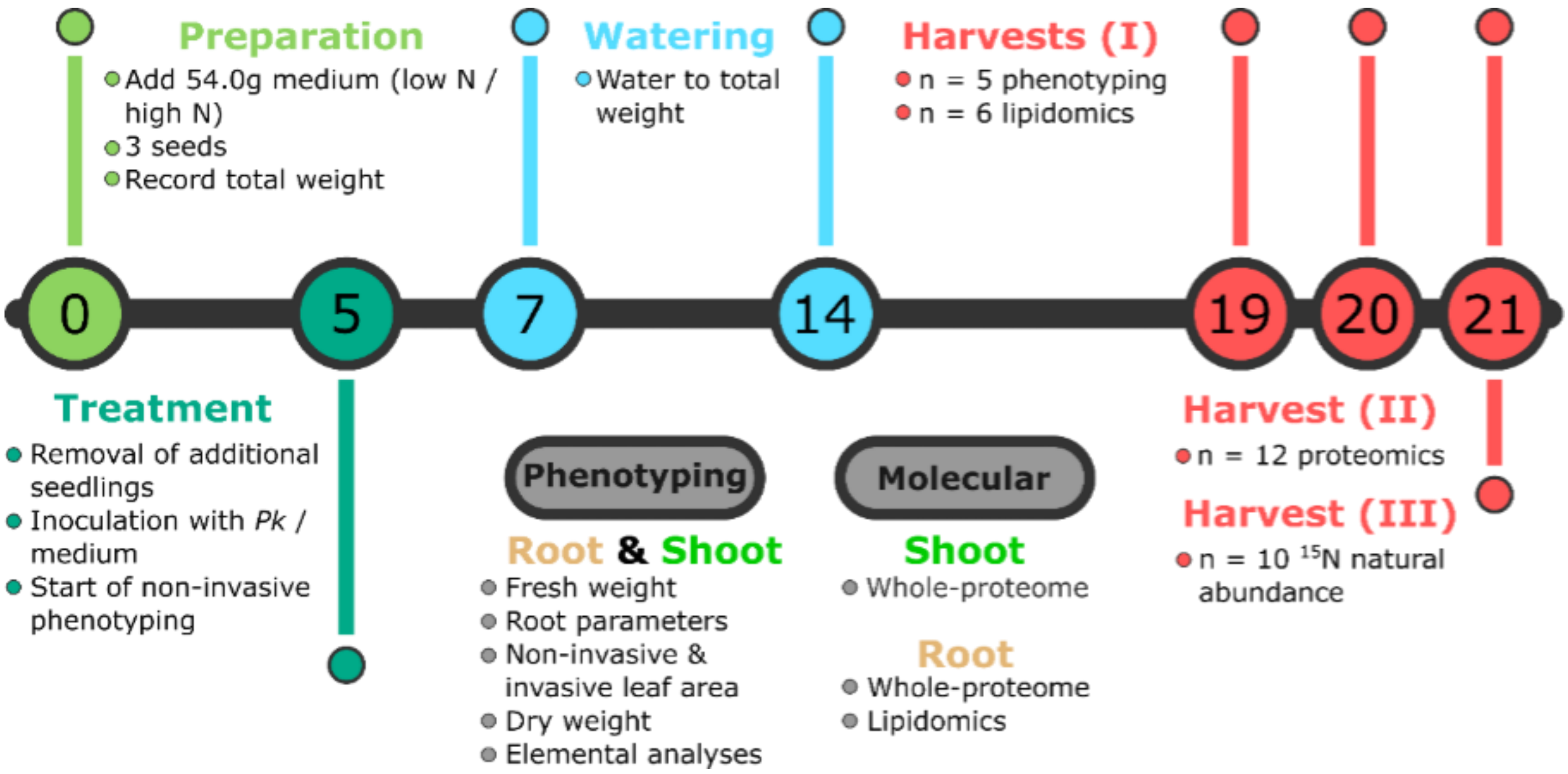

Supplemental Figure S1. **Experimental design and sampling strategy**

Schematic overview of the experimental setup. Numbers in the circles represent days after sowing (DAS). Non-invasive phenotyping was carried out every 2 days after 5 DAS, and on each harvest day. Plants were grown under four different conditions: Low-N, Low-N + *Pk*, High-N, High-N + *Pk*. Three independent experiments were harvested: Harvest (I) Time-series includes consecutive harvests on 19, 20, 21 DAS, with n = 5 plants for phenotyping and n = 6 plants for lipidomics per condition and per timepoint; Harvest (II) at 21 DAS with n = 12 plants for proteomics, and Harvest (III) at 21 DAS with n = 5 plants for $^{15}N/^{14}N$ isotope distribution . Abbreviations: PCV = Plant Cultivation Vessel, *Pk* = *Pseudomonas koreensis.*

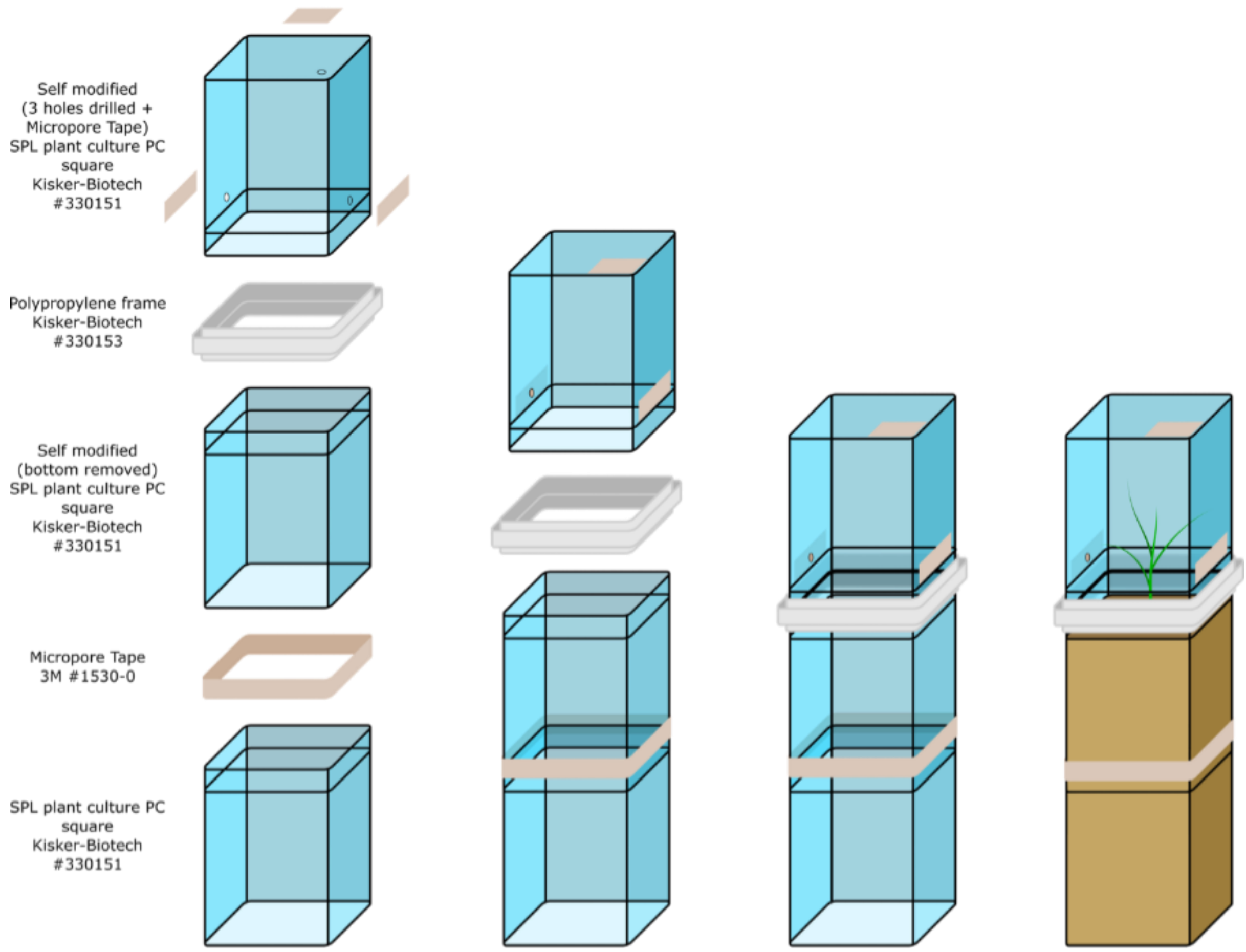

Supplemental Figure S2. Assembly of the Plant Cultivation Vessel (PCV) system

PCV modification and assembly. Three individual Plant Cultivation Vessels (PCV) were stacked on top of each other to obtain a single, closed system. The middle PCV had its bottom sawed off and was stacked on top of the bottommost PCV, using micropore tape (3M) to fix the two containers. The inverted PCV used as a lid was linked to the middle container using the commercially available connector (#330153, Kisker Biotech). For better gas exchange, a total of 3 holes were drilled in the lid.

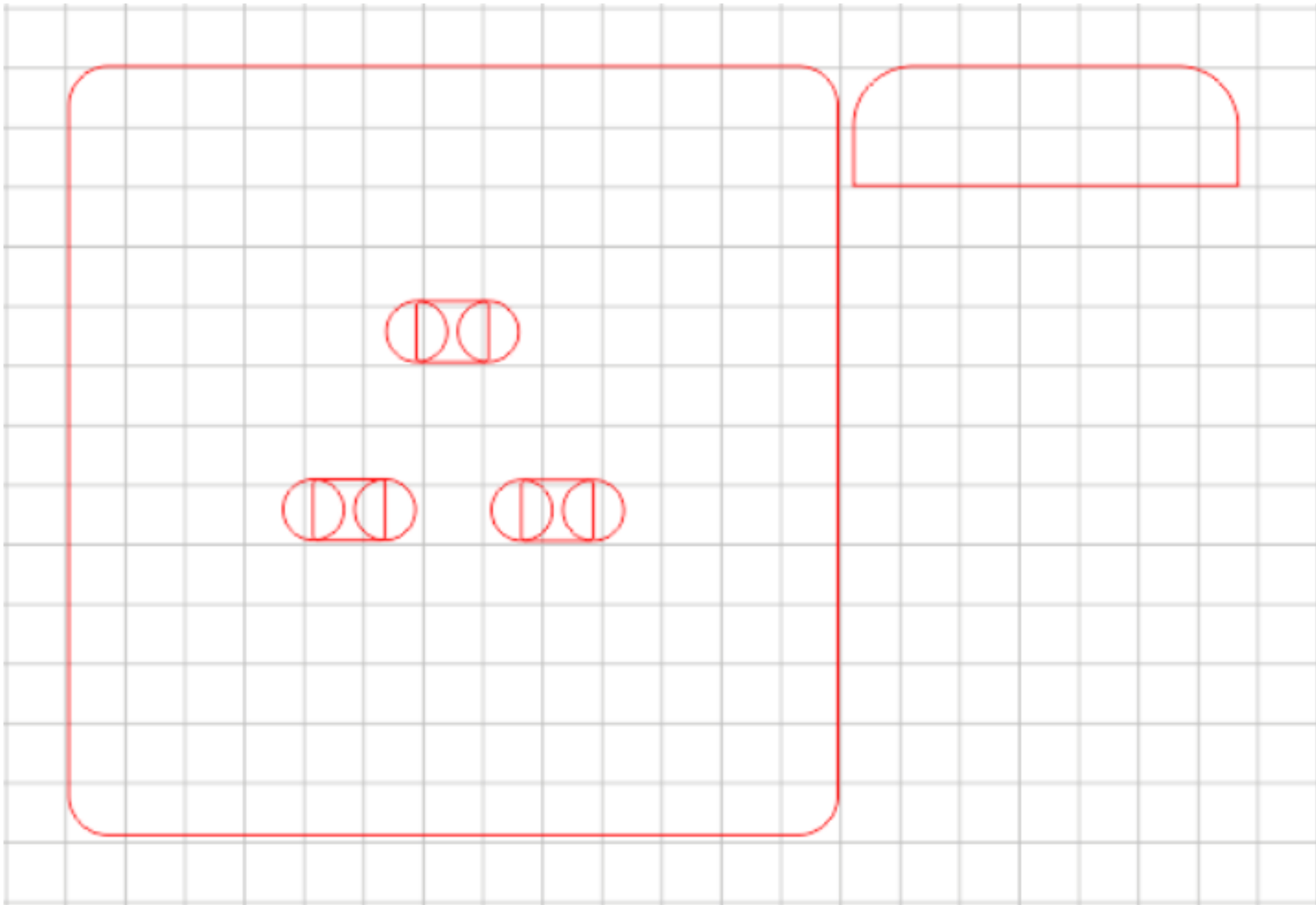

Supplemental Figure S3. Sowing template for standardized seed placement

Sowing template for laser cutter. Left: Base of the sowing template, including 3 holes for fixed seed sowing positions, right: handle, which can be glued to the base for easier operation. The plan for the sowing template, produced by a 3D laser cutter (Trotec, Speedy 100). The sowing template (3 mm thickness) is pressed into the PCV until it is flush with the rim. Then, three holes were poked in the sand using a glass stirring rod, with a stopper at depth of 1.3 cm.

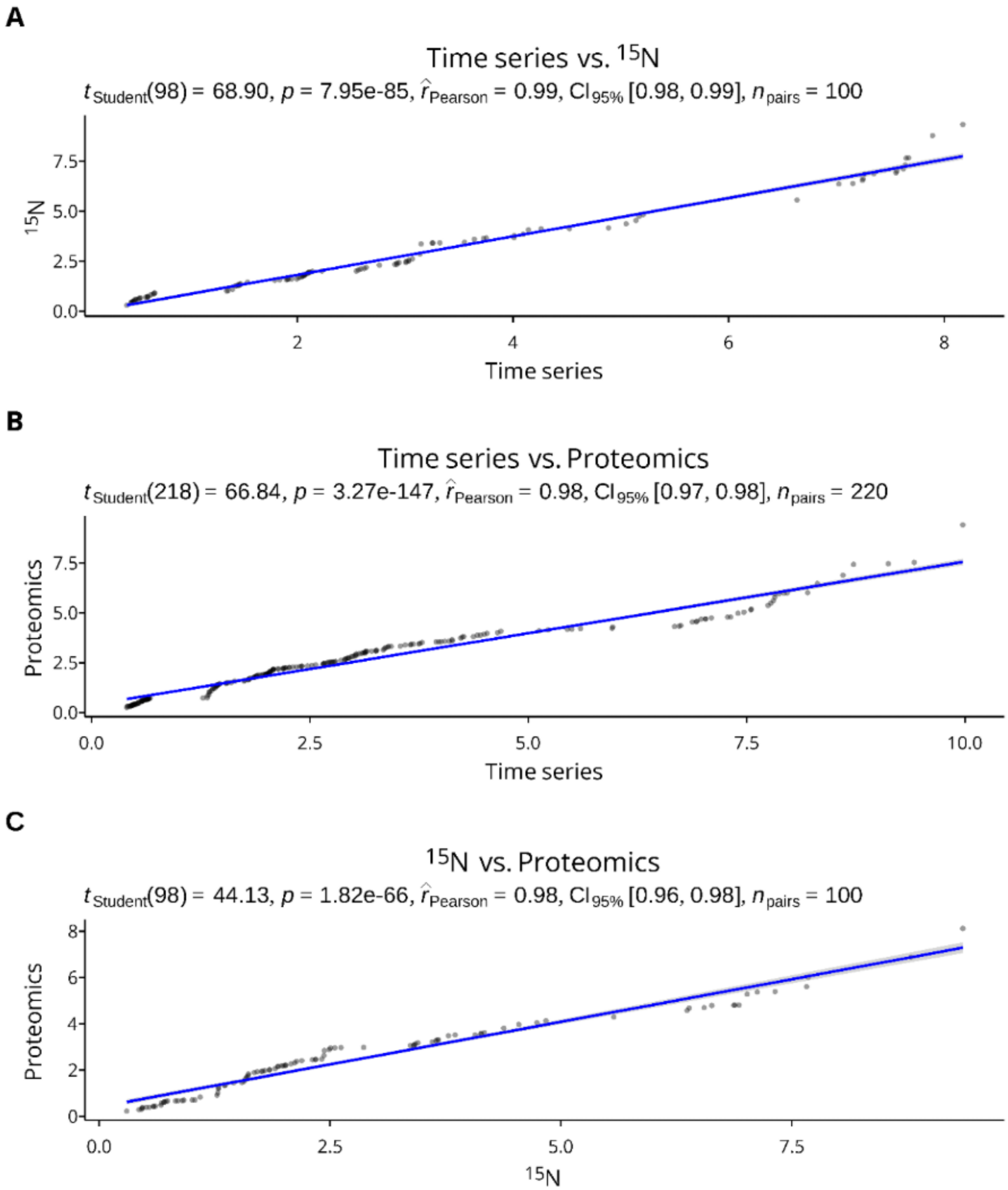

Supplemental Figure S4. Reproducibility of non-invasive shoot phenotyping across experiments

Correlation matrices of projected leaf area between all 3 performed experiments. A) Correlation matrix between time series and ^{15}N (r = 0.99); B) Correlation matrix between time series and Proteomics (r = 0.98); C) Correlation matrix between ^{15}N and proteomics (r = 0.98). Correlations have been calculated using the Pearson correlation coefficient r.

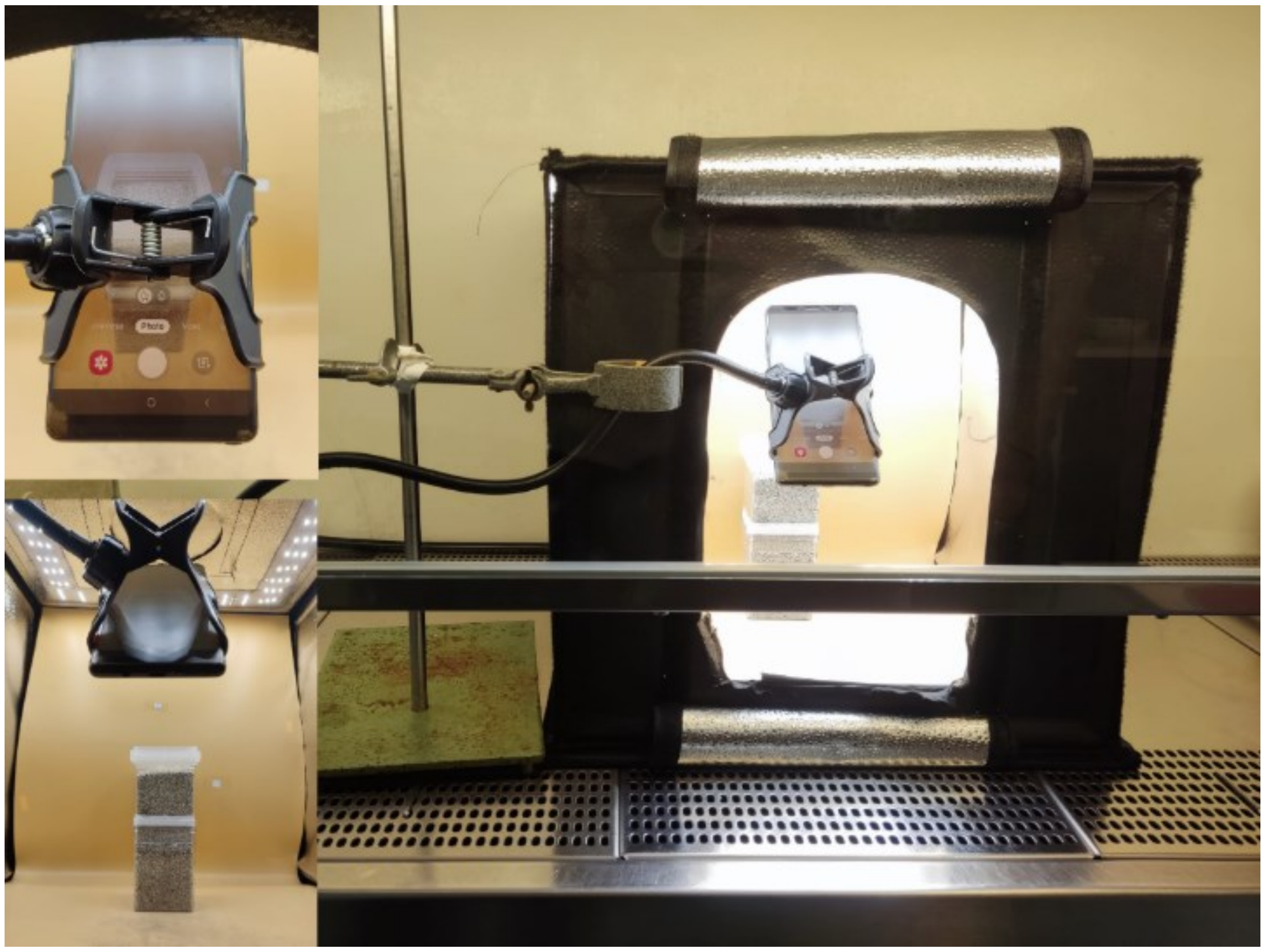

Supplemental Figure S5. Non-invasive shoot phenotyping setup

Non-invasive shoot phenotyping setup inside a biosafety cabinet.

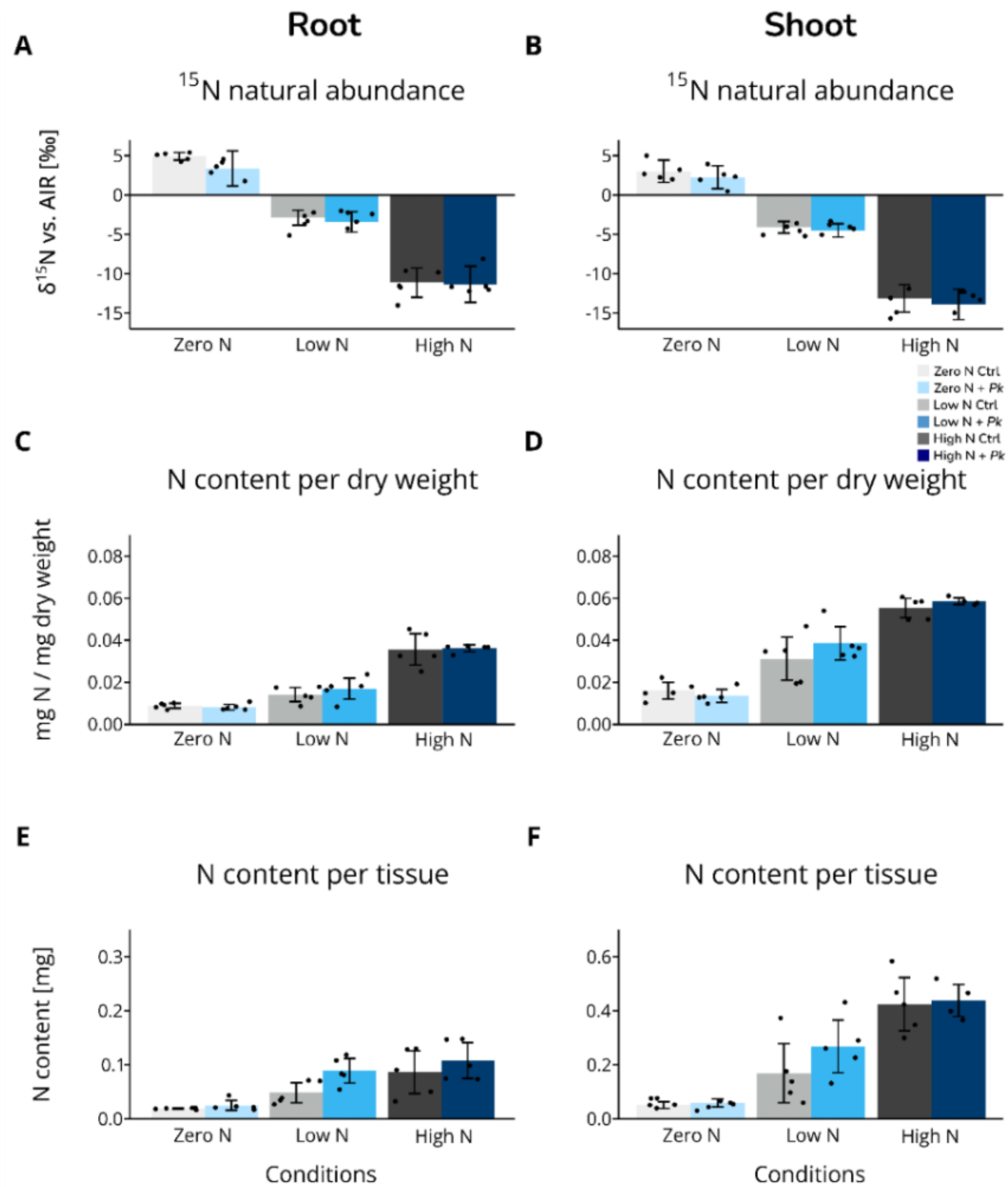

Supplemental Figure S6. Natural abundance δ^{15}N measurements across nitrogen treatments

Measurement of ^{15}N natural abundance in plant tissue at various N availabilities. Data presented are the mean δ^{15}N signatures vs. AIR (n = 5) given as deviation from the medium (δ^{15}N vs. AIR of medium is subtracted) ± standard deviation. Individual replicates are shown as dots on top of their respective bars. Means were compared using ANOVA followed by Tukey honestly significant difference (HSD) test.

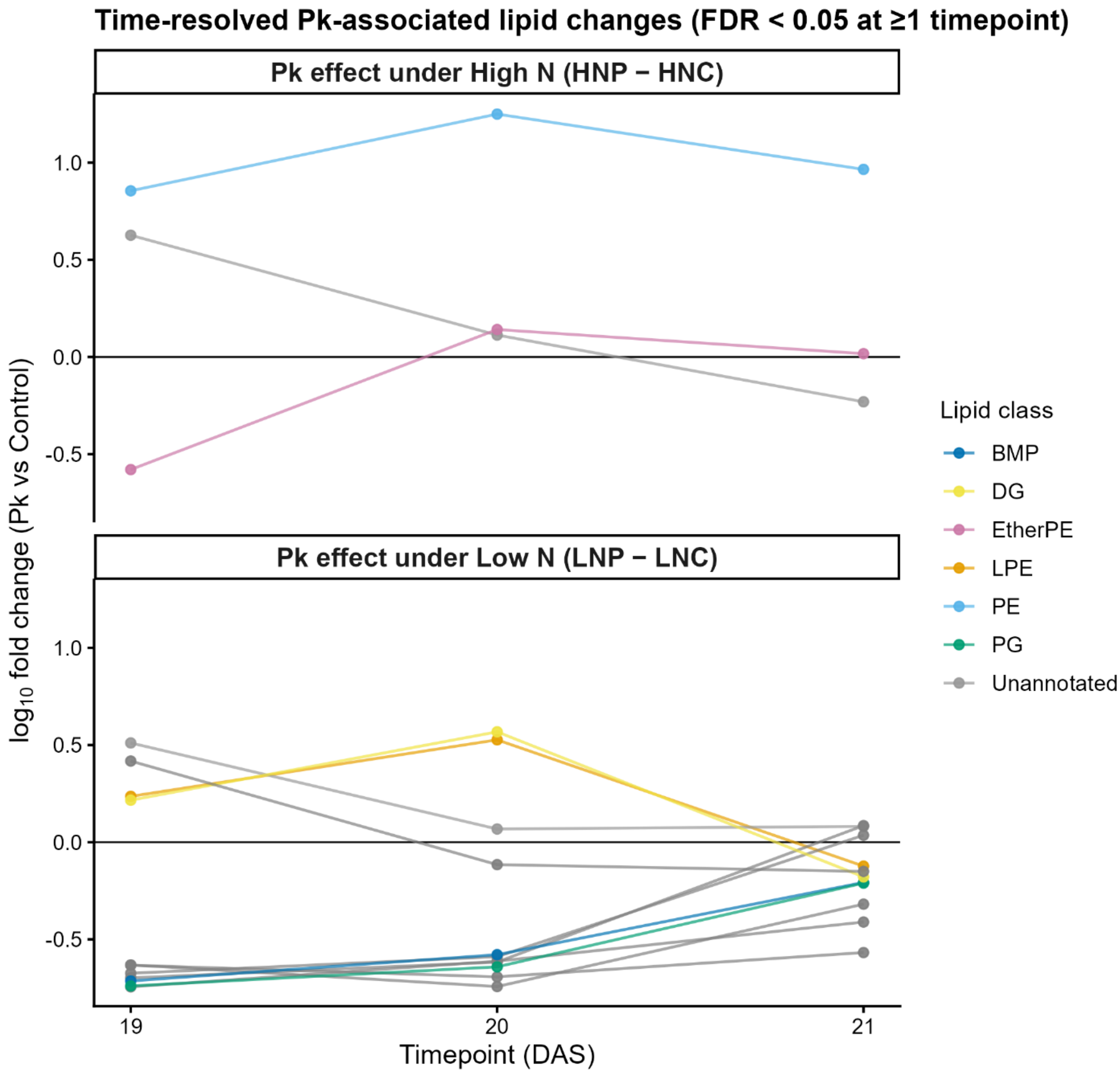

Supplemental Figure S7. Time-resolved lipid responses to *Pseudomonas koreensis* inoculation

Time-resolved Pk-associated lipid changes in roots. Shown are $\log_{10}$ fold changes derived from linear models for lipid features that exhibited a statistically significant response to Pk inoculation (FDR < 0.05) at at least one time point. Lipid features are plotted across 19, 20, and 21 days after sowing (DAS) for the contrasts Low N + Pk vs. Low N control (LNP − LNC) and High N + Pk vs. High N control (HNP − HNC). Each line represents an individual lipid feature, grouped by Alignment ID; points indicate estimated effect sizes at each time point. Lipid classes are indicated by color where available; unannotated features are shown in grey. Statistics corresponding to all plotted features are provided in Supplemental Table S6. Abbreviaitons: Bis[monoacylglycero]phosphates - BMP; diacylglycerols - DG; lysophospholipids - LPE ; phosphatidylethanolamine- PE,; Phosphatidylgylcerols- PG.

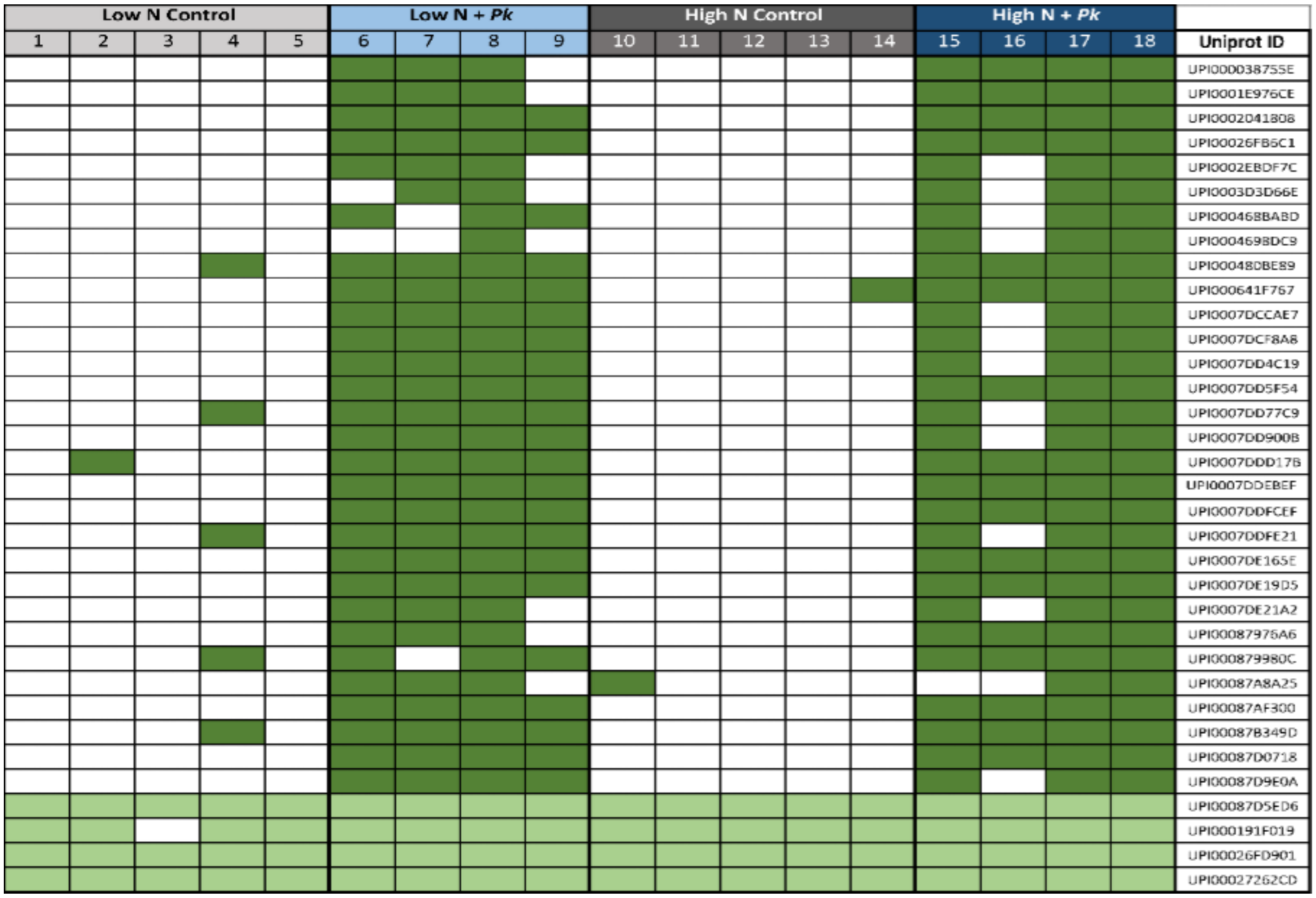

Supplemental Figure S8. Detection of *Pseudomonas koreensis* proteins across samples

Presence or absence of *Pk*-derived proteins across individual samples. Dark green indicates detection of *Pk*-specific proteins; light green indicates conserved bacterial proteins present in all samples.

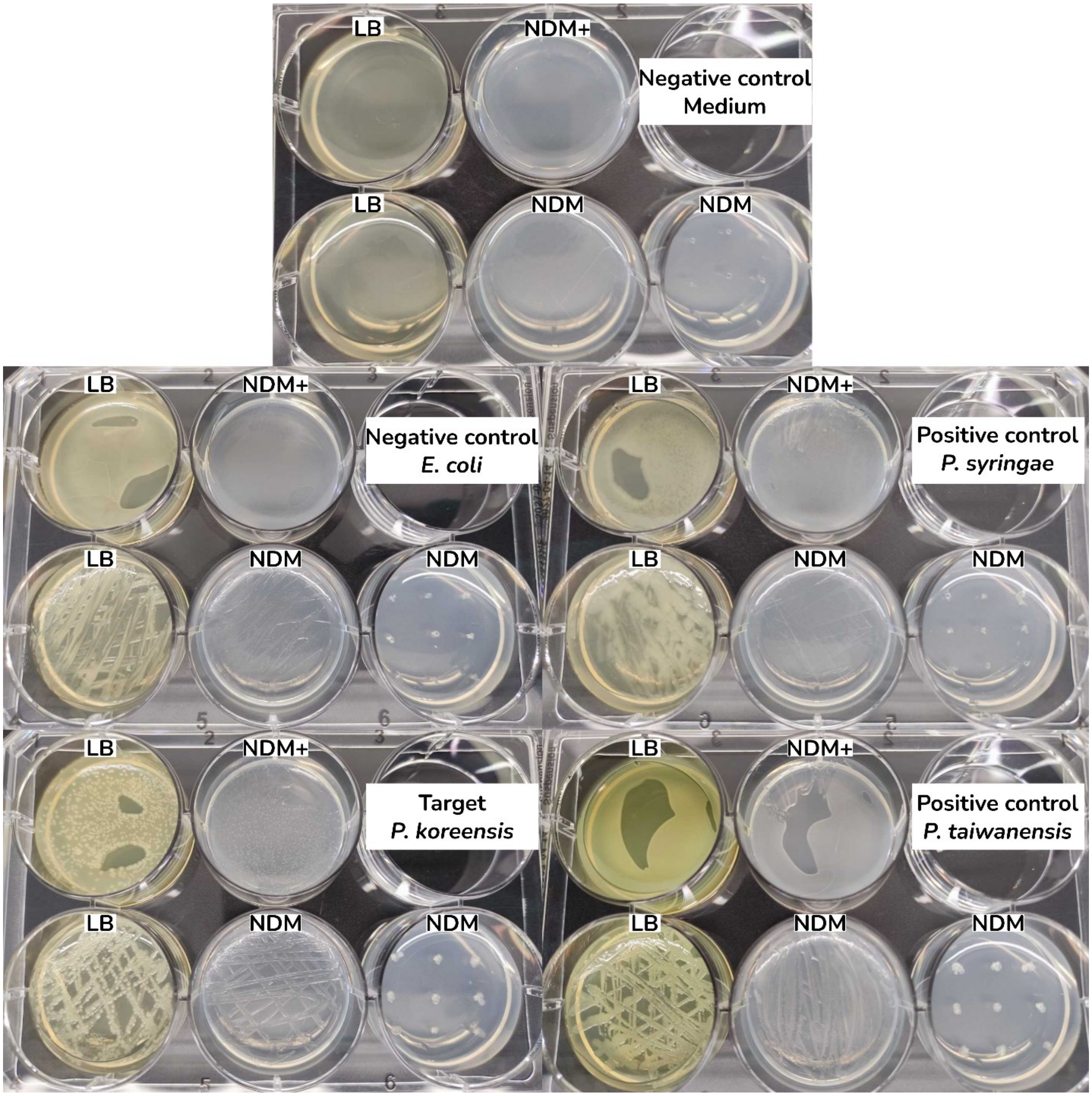

Supplemental Figure S9. NDM bacteria assay. Representative 6-well plates from the deficient media plate assay. Wells 1 and 2 contain LB medium; well 3 contains nitrogen-free medium supplemented with N (NFM+); wells 4 and 5 contain nitrogen-free medium (NFM). Well 4 was surface inoculated with bacteria. In well 5, bacteria were introduced by puncturing the agar.

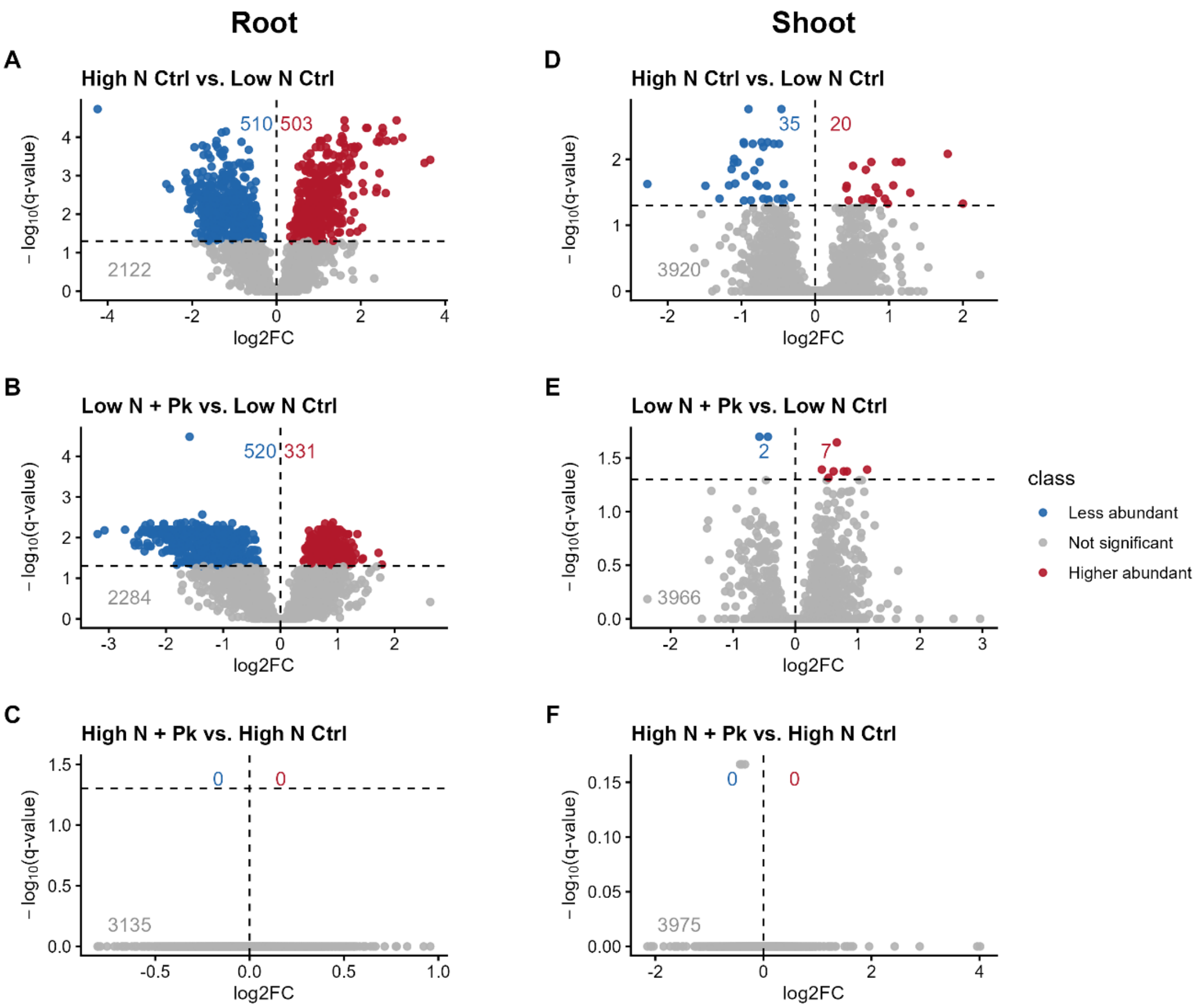

Supplemental Figure S10. Differential protein abundance in roots and shoots

Volcano plots of differential protein abundance in roots and shoots. (A–C) Root comparisons: (A) High-N Control vs. Low-N Control, (B) Low-N + *Pseudomonas koreensis* vs. Low-N Control, and (C) High-N + *P. koreensis* vs. High-N Control. (D–F) Corresponding comparisons in shoots. Analyses include only proteins quantified with 100% valid values within each dataset. The y-axis shows $-\log_{10}$-transformed *p*-values from Tukey's HSD post hoc tests, adjusted for multiple testing using the Benjamini–Hochberg procedure (FDR < 0.05). No fold-change cutoff was applied ($\log_2 FC = 0$). Numbers indicate the count of proteins in each category: less abundant (red), not significant (grey), and higher abundant (blue), matching point colors.

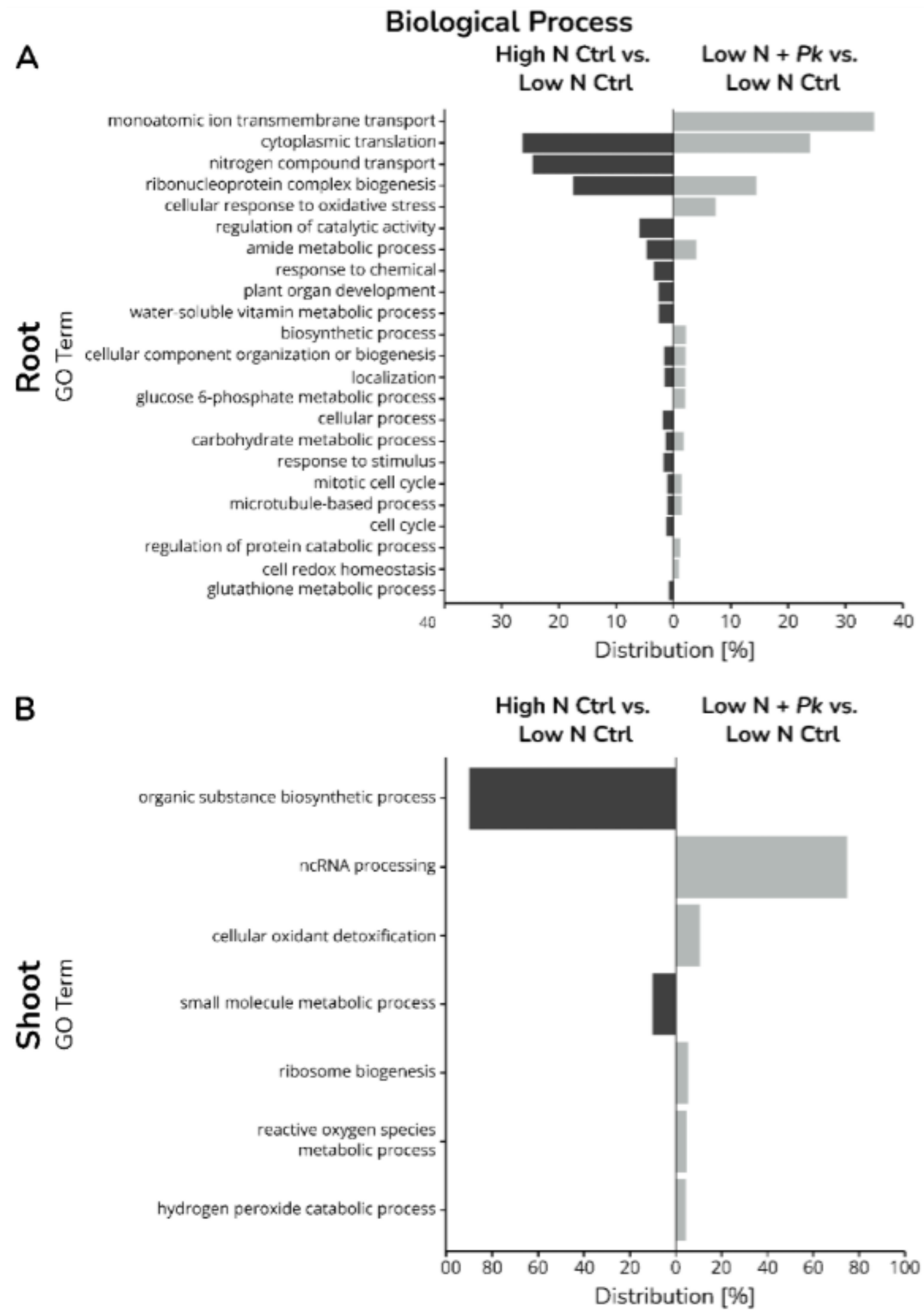

Supplemental Figure S11. Enriched biological process GO terms associated with nitrogen and Pk responses

GO term analyses of Biological Processes. Only statistical significant proteins were imported into PantherDB statistical enrichment test (Mi et al., 2019), followed by REVIGO (Supek et al., 2011). Log10 (p-values) were used to calculate the distribution in percent (x-axis) of GO terms (y-axis). A) Root biological process GO-Terms; B) Shoot biological GO-Terms.

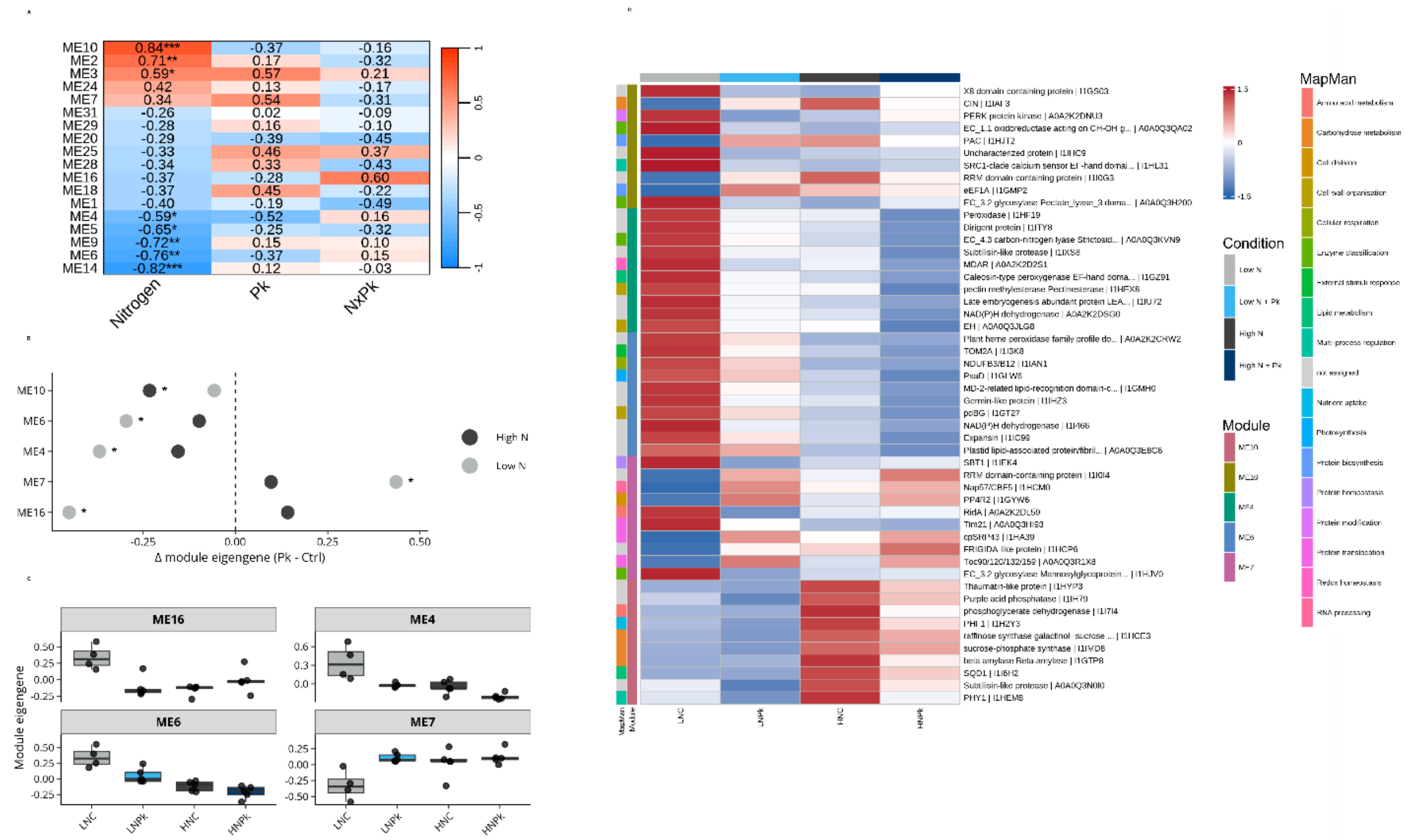

Supplemental Figure S12. WGCNA module expression patterns in shoot tissue

Weighted gene co-expression network analysis (WGCNA) of the shoot proteome under contrasting nitrogen and *Pseudomonas koreensis* (Pk) treatments. The heatmap shows z-scored mean protein abundances for hub proteins (top-ranked by absolute module membership, kME) from selected shoot modules across Low nitrogen control (LNC), Low nitrogen + Pk (LNPk), High nitrogen control (HNC), and High nitrogen + Pk (HNPk) conditions. Proteins are grouped by module assignment (left annotation bar) and functionally annotated using MapMan Level-1 categories (left annotation bar). Protein abundance values were averaged across biological replicates within each condition and scaled by row (protein) to highlight relative expression differences across treatments. This figure complements the shoot module–trait correlations reported in Supplemental Table S6 and the hub protein lists provided in Supplemental Table S7, S8.

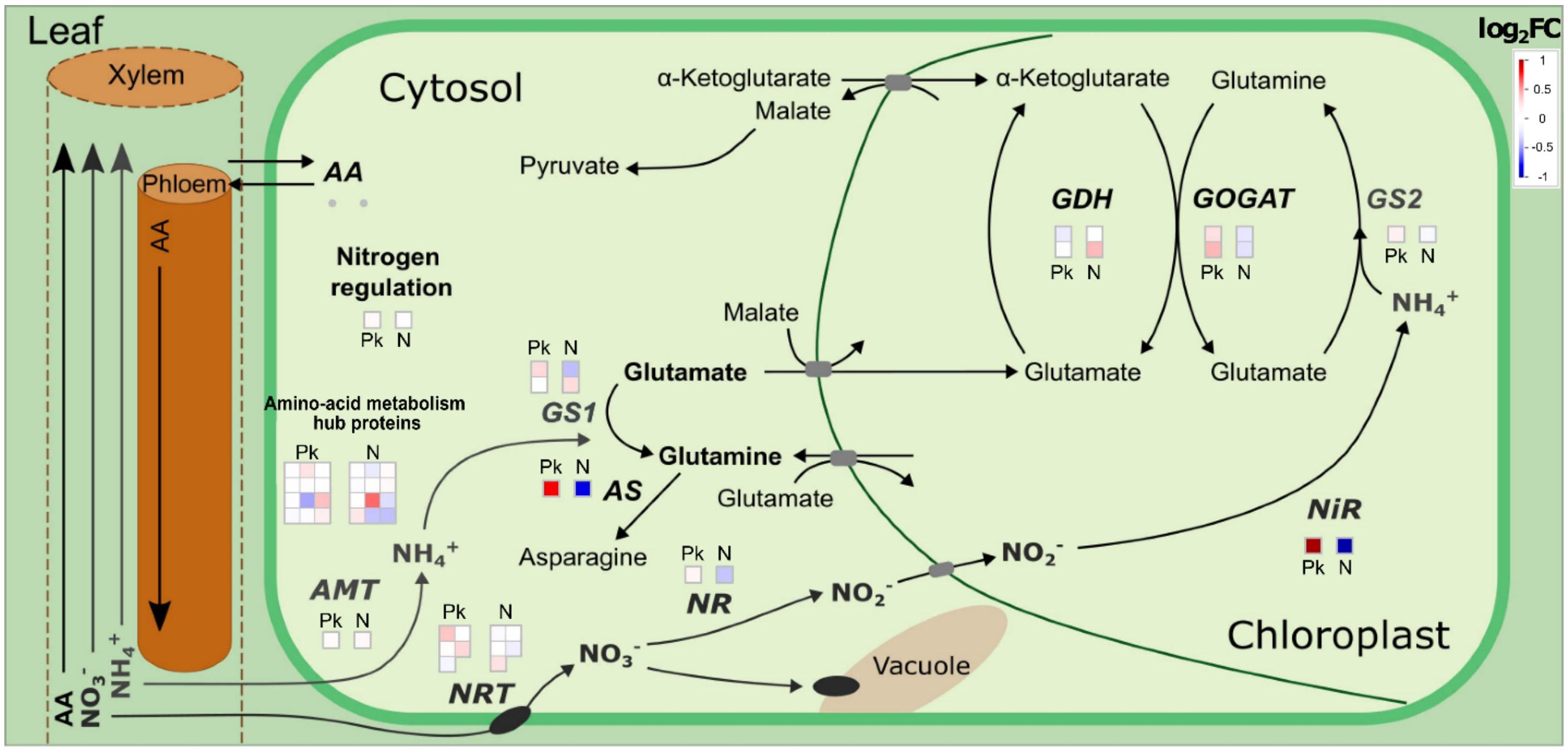

Supplemental Figure S13. Schematic representation of central nitrogen and amino-acid metabolism in shoots, annotated with WGCNA-identified hub proteins quantified across all samples. Colored squares indicate log_2 fold changes relative to low-nitrogen control (LNC), shown as Low N + Pk – LNC (Pk effect) and High N control – LNC (nitrogen effect). Only root proteins meeting the 100% data-completeness criterion are shown. Pathway structure is based on MapMan (version 3.5.1R2; Thimm et al., 2004) nitrogen uptake and assimilation modules with custom modifications; mapping files were generated using Mercator4 (Schwacke et al., 2019). Mapped proteins, log_2 fold changes, and statistical significance are provided in Supplemental Table S2. Abbreviations: AA, amino acids; AMT, ammonium transporter; AS, asparagine synthase; GDH, glutamate dehydrogenase; GOGAT, glutamine oxoglutarate aminotransferase; GS1, cytosolic glutamine synthetase; GS2, plastidial glutamine synthetase; NiR, nitrite reductase; NR, nitrate reductase; NRT, nitrate transporter.

4. Supplemental Tables

Supplemental Table S1. Integrated summary of phenotypic, elemental, proteomic, and lipidomic responses to *Pseudomonas koreensis* under contrasting nitrogen conditions at 21 DAS

Summary of treatment effects of *Pseudomonas koreensis* (Pk) inoculation under low- and high-nitrogen (N) conditions at 21 days after sowing (DAS). Arrows indicate the direction and relative magnitude of statistically supported effects of Pk inoculation compared to the corresponding uninoculated control within each nitrogen regime (Low-N + Pk vs. Low-N Ctrl; High-N + Pk vs. High-N Ctrl). Double arrows (↑↑) denote stronger effects relative to single arrows (↑), while ↔ indicates no significant change.

Phenotypic and elemental traits are based on two-way ANOVA with nitrogen availability and Pk inoculation as factors, followed by Tukey's HSD post hoc tests ($\alpha = 0.05$). Proteomic responses summarize system-level patterns derived from weighted gene co-expression network analysis (WGCNA), including shifts in module eigengene behavior and enrichment of nitrogen- and amino-acid–related functional categories. Lipidomic responses reflect multivariate analyses (PCA/HCA) and per-feature statistical testing across time points. No evidence for in planta biological nitrogen fixation was detected under any condition.

Category	Trait / Process	Pk effect under Low N (Low-N + Pk vs Low-N)	Pk effect under High N (High-N + Pk vs High-N)
**Growth**	Shoot biomass	↑↑	↔
	Root biomass	↑	↔
**Carbon (absolute)**	Shoot C (total content)	↔	↔
	Root C (total content)	↔	↔
**Carbon (relative)**	Shoot C concentration	↔	↔
	Root C concentration	↓	↔
**Nitrogen (absolute)**	Shoot N (total content)	↑↑ (to High-N–like levels)	↔
	Root N (total content)	↑	↔
**Nitrogen (relative)**	Shoot N concentration	↑	↔
	Root N concentration	↔	↔
**Proteomics (system-level)**	Global eigengene organization	Converges toward High-N state	↔

	N / amino-acid metabolism modules	↑	↔
	Regulatory / signaling modules	Conditional modulation	↔
**Lipidomics**	Global lipidome (PCA/HCA)	Modest, transient shift (19 DAS)	↔
	Individual lipid species	Few significant changes	↔
**BNF evidence**	In-planta N fixation	No evidence detected	Not applicable

Supplemental Table S2. Summary statistics and post hoc analyses for plant phenotypic traits and tissue carbon and nitrogen content under contrasting nitrogen and bacterial treatments.

The table reports means, sample sizes (n), and adjusted p-values for plant phenotypic traits and tissue carbon (C) and nitrogen (N) contents measured under Low and High nitrogen conditions with or without *Pseudomonas* (Pk) inoculation. Data were analysed separately for each time point and tissue using two-way ANOVA with Nitrogen and Bacterium as fixed factors, followed by Tukey's HSD post hoc tests. For Figure 2, significance refers to pairwise comparisons between Pk and Ctrl within each nitrogen regime at 21 days after sowing (DAS). Relative C and N contents are expressed per unit dry weight, while absolute contents are reported per plant. Exact adjusted p-values are provided; asterisks in figures denote $p < 0.05$, $p < 0.01$, and $p < 0.001$.

- ➔ Supplemental_Table_S2_Phenotypes.xlsx

Supplemental Table S3. Proteomic coverage and numbers of differentially abundant proteins across treatments

Summary of proteomic coverage and differential protein abundance in roots and shoots. Shown are the numbers of identified proteins, proteins quantified with complete data across all conditions (100% valid values), and proteins exhibiting significant pairwise differences between treatment contrasts. Pairwise statistical significance was determined using Tukey's Honestly Significant Difference (HSD) tests followed by Benjamini–Hochberg false discovery rate correction applied across proteins (tukey_q_fdr < 0.05). Permutation-based multi-sample ANOVA results generated in Perseus are provided in Supplemental Table S4.

Tissue	**Identified proteins**	**Proteins with complete data (all conditions)**	**High N Ctrl vs Low N Ctrl (FDR < 0.05)**	**Low-N + *Pk* vs. Low-N Ctrl**	**High-N + *Pk* vs. High-N Ctrl**

				(Tukey's HSD FDR <0.05)	(Tukey's HSD FDR <0.05)
**Root**	5660	3135	1013	851	0
**Shoot**	6656	3975	55	9	0

Supplemental Table S4. Differential protein abundance statistics in roots and shoots

Differential protein abundance statistics for root and shoot proteomes across nitrogen and *Pseudomonas koreensis* (Pk) treatments. The table reports $\log_2$ fold changes for all pairwise contrasts, raw Tukey's HSD p-values, and corresponding significance codes for reference. Statistical significance of pairwise comparisons was determined using Benjamini–Hochberg–adjusted q-values calculated across proteins within each contrast (*tukey_q_fdr*).

Permutation-based q-values derived from multi-sample ANOVA in Perseus (*anova_q_permutation*) are additionally reported to indicate overall treatment effects at the protein level. Functional annotation using MapMan (BIN, BINCODE, and BIN name) is provided to facilitate biological interpretation. Only proteins quantified with 100% valid values within the respective tissue dataset were considered.

➔ STable4_DAP_Roots_Shoots_with_BHq.xlsx

Supplemental Table S5. Lipid species with significant abundance changes across time points

List of lipid features showing statistically significant changes in abundance in at least one time point following *Pseudomonas koreensis* (Pk) inoculation under low-nitrogen conditions. The table reports lipid annotations, mass-to-charge ratios, retention times, time points, and pairwise contrasts with corresponding $\log_{10}$ fold changes and Tukey's HSD statistics.

Statistical testing was performed separately for each time point using two-way ANOVA followed by Tukey's HSD post hoc tests. Reported false discovery rates (FDR) correspond to Benjamini–Hochberg correction applied across lipid features within each time point and contrast. Lipid annotations reflect MS-DIAL output and include both annotated and unannotated features. This table underlies the time-resolved lipid abundance profiles shown in Supplemental Figure S7.

➔ STable5_Lipids_ANOVA_Tukey_byTimepoint_full.xlsx

Supplemental Table S6. Module-trait associations

Correlation analysis between WGCNA module eigengenes and experimental traits. For each module–trait pair, the Pearson correlation coefficient (Cor), nominal P-value (P), and Benjamini–Hochberg–adjusted false discovery rate (FDR) are reported. Traits include nitrogen status (Nitrogen), bacterial inoculation with *Pseudomonas koreensis* (Pk), and their interaction (NxPk). Analyses were performed separately for root and shoot proteomes.

➔ STableS6_Module_Trait_Correlations

Supplemental Table S7. Hub proteins per module

Top hub proteins for each WGCNA module, ranked by absolute module membership (kME). Hub proteins are defined as the top-k proteins showing the strongest correlation with the corresponding module eigengene. Functional annotations include MapMan Level-1 bin (BIN), full MapMan BINCODE, MapMan BIN name, and Mercator protein annotations (Mercator_Name and Mercator_Description) to facilitate pathway-level and protein-level interpretation. Tables are provided separately for root and shoot networks.

➔ STableS7_HubProteins_PerModule

Supplemental Table S8. Full module protein membership

Complete list of proteins assigned to each WGCNA module. For each protein, module membership strength (kME_inModule) is reported together with functional annotations derived from MapMan and Mercator. MapMan annotations include Level-1 bin (BIN), full BINCODE, and BIN name, whereas Mercator annotations provide protein-level functional descriptions. This table represents the full protein universe used for network construction and downstream enrichment analyses.

➔ STableS8_Full_Module_Protein_Membership.xlsx
