# Supplementary material for: Systems‐Level Plant Responses Reveal Pseudomonas‐Mediated Growth Promotion in Brachypodium Under Nitrogen Limitation
Source: Plant Cell Environ. 2026 May 25;49(9):6396–412. doi: 10.1111/pce.70615 (PMC13436448; doi:10.1111/pce.70615)
Supplement: Supplementary file 2 [file PCE-49-6396-s006.docx]

**Supplemental Table S1. Integrated summary of phenotypic, elemental, proteomic, and lipidomic responses to *Pseudomonas koreensis* under contrasting nitrogen conditions at 21 DAS**

Summary of treatment effects of *Pseudomonas koreensis* (Pk) inoculation under low- and high-nitrogen (N) conditions at 21 days after sowing (DAS). Arrows indicate the direction and relative magnitude of statistically supported effects of Pk inoculation compared to the corresponding uninoculated control within each nitrogen regime (Low-N + Pk vs. Low-N Ctrl; High-N + Pk vs. High-N Ctrl). Double arrows (↑↑) denote stronger effects relative to single arrows (↑), while ↔ indicates no significant change.

Phenotypic and elemental traits are based on two-way ANOVA with nitrogen availability and Pk inoculation as factors, followed by Tukey’s HSD post hoc tests (α = 0.05). Proteomic responses summarize system-level patterns derived from weighted gene co-expression network analysis (WGCNA), including shifts in module eigengene behavior and enrichment of nitrogen- and amino-acid–related functional categories. Lipidomic responses reflect multivariate analyses (PCA/HCA) and per-feature statistical testing across time points. No evidence for in planta biological nitrogen fixation was detected under any condition.

| **Category** | **Trait / Process** | **Pk effect under Low N (Low-N + Pk vs Low-N)** | **Pk effect under High N (High-N + Pk vs High-N)** |
| --- | --- | --- | --- |
| **Growth** | Shoot biomass | ↑↑ | ↔ |
|  | Root biomass | ↑ | ↔ |
| **Carbon (absolute)** | Shoot C (total content) | ↔ | ↔ |
|  | Root C (total content) | ↔ | ↔ |
| **Carbon (relative)** | Shoot C concentration | ↔ | ↔ |
|  | Root C concentration | ↓ | ↔ |
| **Nitrogen (absolute)** | Shoot N (total content) | ↑↑ (to High-N–like levels) | ↔ |
|  | Root N (total content) | ↑ | ↔ |
| **Nitrogen (relative)** | Shoot N concentration | ↑ | ↔ |
|  | Root N concentration | ↔ | ↔ |
| **Proteomics (system-level)** | Global eigengene organization | Converges toward High-N state | ↔ |
|  | N / amino-acid metabolism modules | ↑ | ↔ |
|  | Regulatory / signaling modules | Conditional modulation | ↔ |
| **Lipidomics** | Global lipidome (PCA/HCA) | Modest, transient shift (19 DAS) | ↔ |
|  | Individual lipid species | Few significant changes | ↔ |
| **BNF evidence** | In-planta N fixation | No evidence detected | Not applicable |
