# Supplementary material for: Systems‐Level Plant Responses Reveal Pseudomonas‐Mediated Growth Promotion in Brachypodium Under Nitrogen Limitation
Source: Plant Cell Environ. 2026 May 25;49(9):6396–412. doi: 10.1111/pce.70615 (PMC13436448; doi:10.1111/pce.70615)
Supplement: Supplementary file 3 [file PCE-49-6396-s007.docx]

**Supplemental Table S3. Proteomic coverage and numbers of differentially abundant proteins across treatments**

Summary of proteomic coverage and differential protein abundance in roots and shoots. Shown are the numbers of identified proteins, proteins quantified with complete data across all conditions (100% valid values), and proteins exhibiting significant pairwise differences between treatment contrasts. Pairwise statistical significance was determined using Tukey’s Honestly Significant Difference (HSD) tests followed by Benjamini–Hochberg false discovery rate correction applied across proteins (tukey_q_fdr < 0.05). Permutation-based multi-sample ANOVA results generated in Perseus are provided in Supplemental Table S4.

| Tissue | **Identified proteins** | **Proteins with complete data (all conditions)** | **High N Ctrl vs Low N Ctrl (FDR < 0.05)** | **Low-N + *Pk* vs. Low-N Ctrl**  **(Tukey’s HSD FDR <0.05)** | **High-N + *Pk* vs. High-N Ctrl**  **(Tukey’s HSD FDR <0.05)** |
| --- | --- | --- | --- | --- | --- |
| **Root** | 5660 | 3135 | 1013 | 851 | 0 |
| **Shoot** | 6656 | 3975 | 55 | 9 | 0 |
